# Supplementary figures and images for: Vascular plant biodiversity of the lower Coppermine River valley and vicinity (Nunavut, Canada): an annotated checklist of an Arctic flora
Source: PeerJ. 2017 Jan 31;5:e2835. doi: 10.7717/peerj.2835 (PMC5300018; doi:10.7717/peerj.2835)

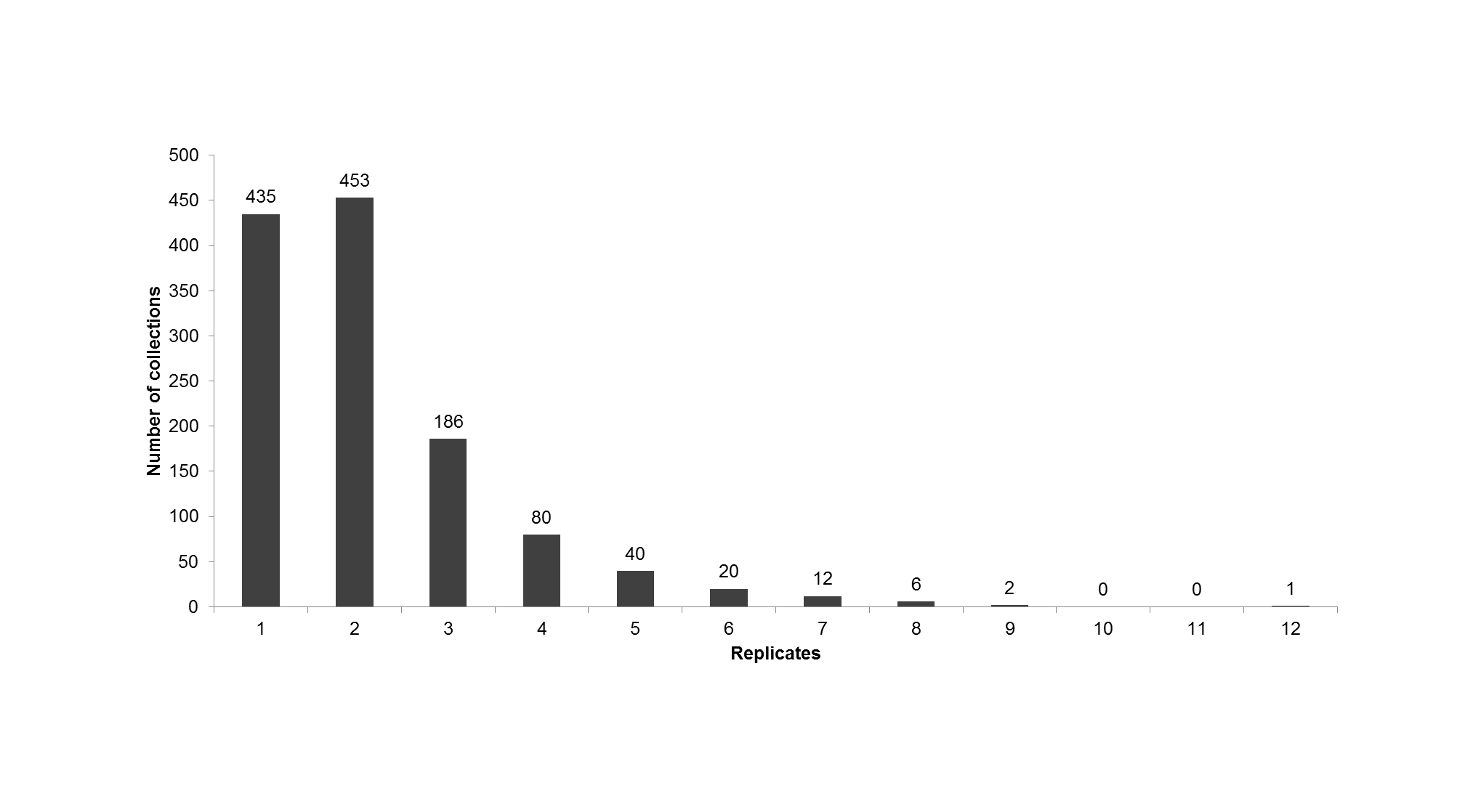

Supplement: Supplemental Information 1 [file peerj-05-2835-s001.png]

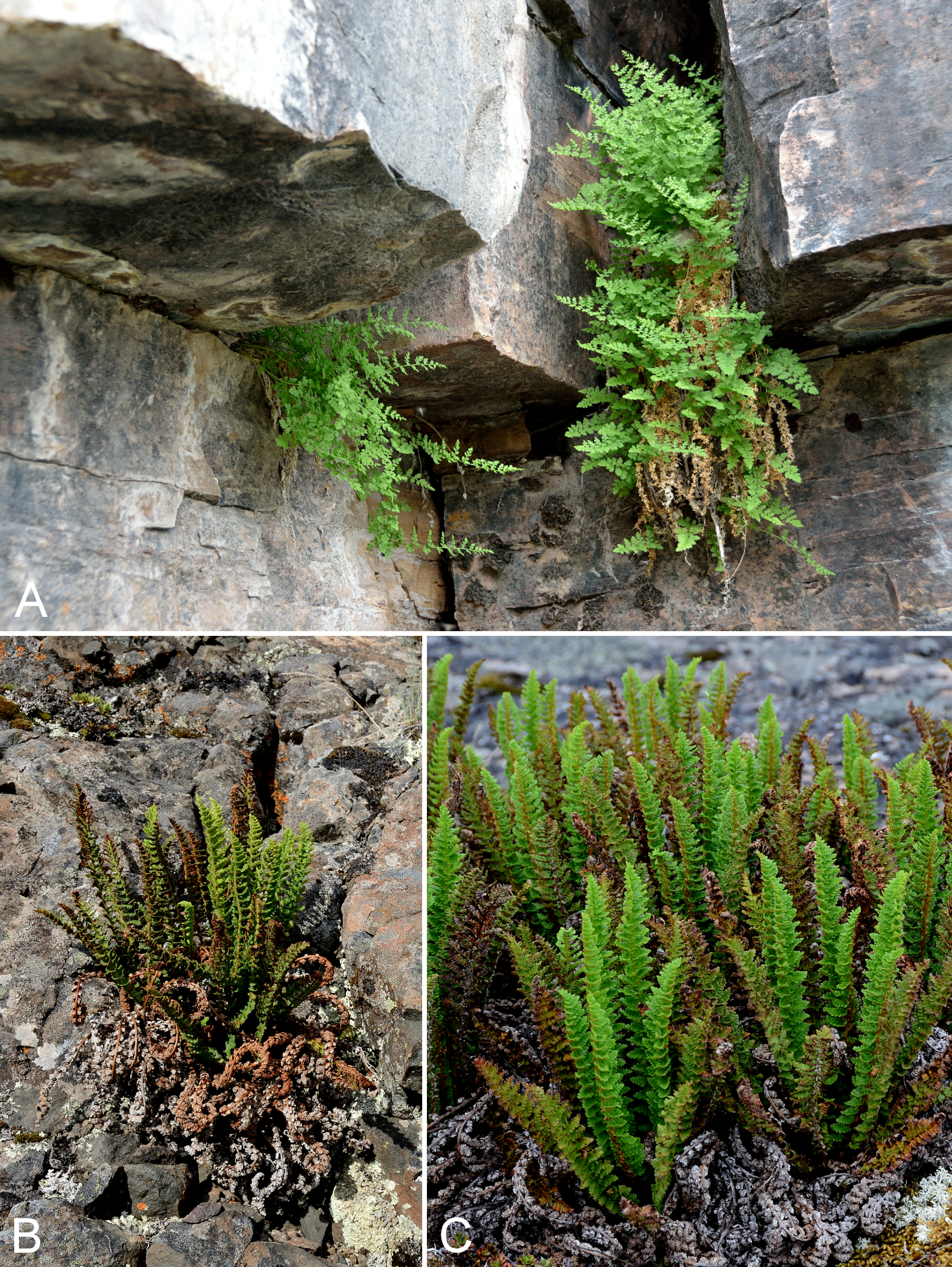

Supplement: Supplemental Information 2 — Cystopteris fragilis: (A) habit, Saarela et al. 3577. Dryopteris fragrans: (B) habitat, Saarela et al. 3434. (C) habit, Saarela et al. 3934. Photographs by R. D. Bull. [file peerj-05-2835-s002.png]

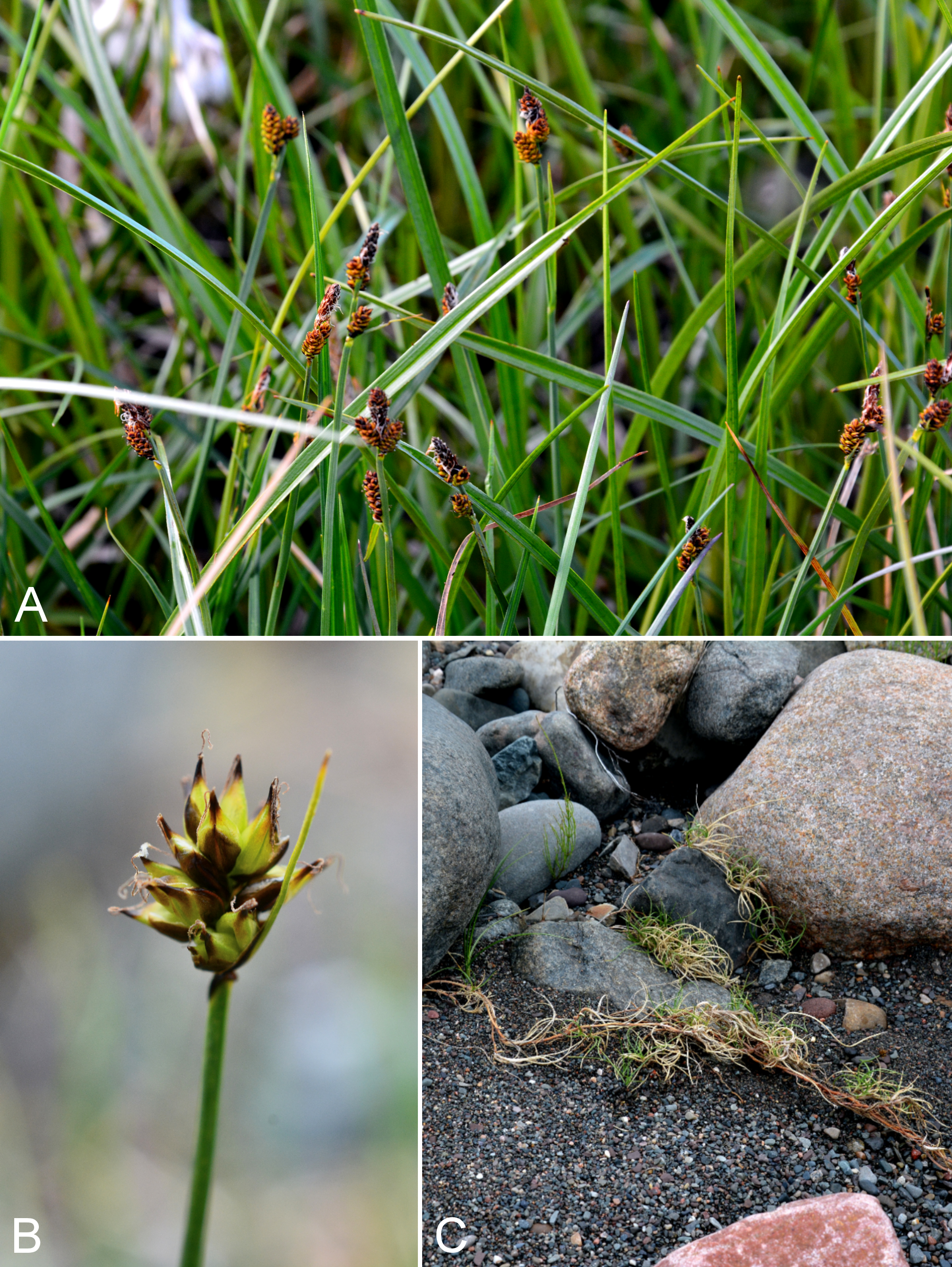

Supplement: Supplemental Information 3 — Carex marina: (A) habit, Saarela et al. 4095. Carex maritima: (B) inflorescence, Saarela et al. 4117. (C) habit, Saarela et al. 4117. Photographs by P.C. Sokoloff. [file peerj-05-2835-s003.png]

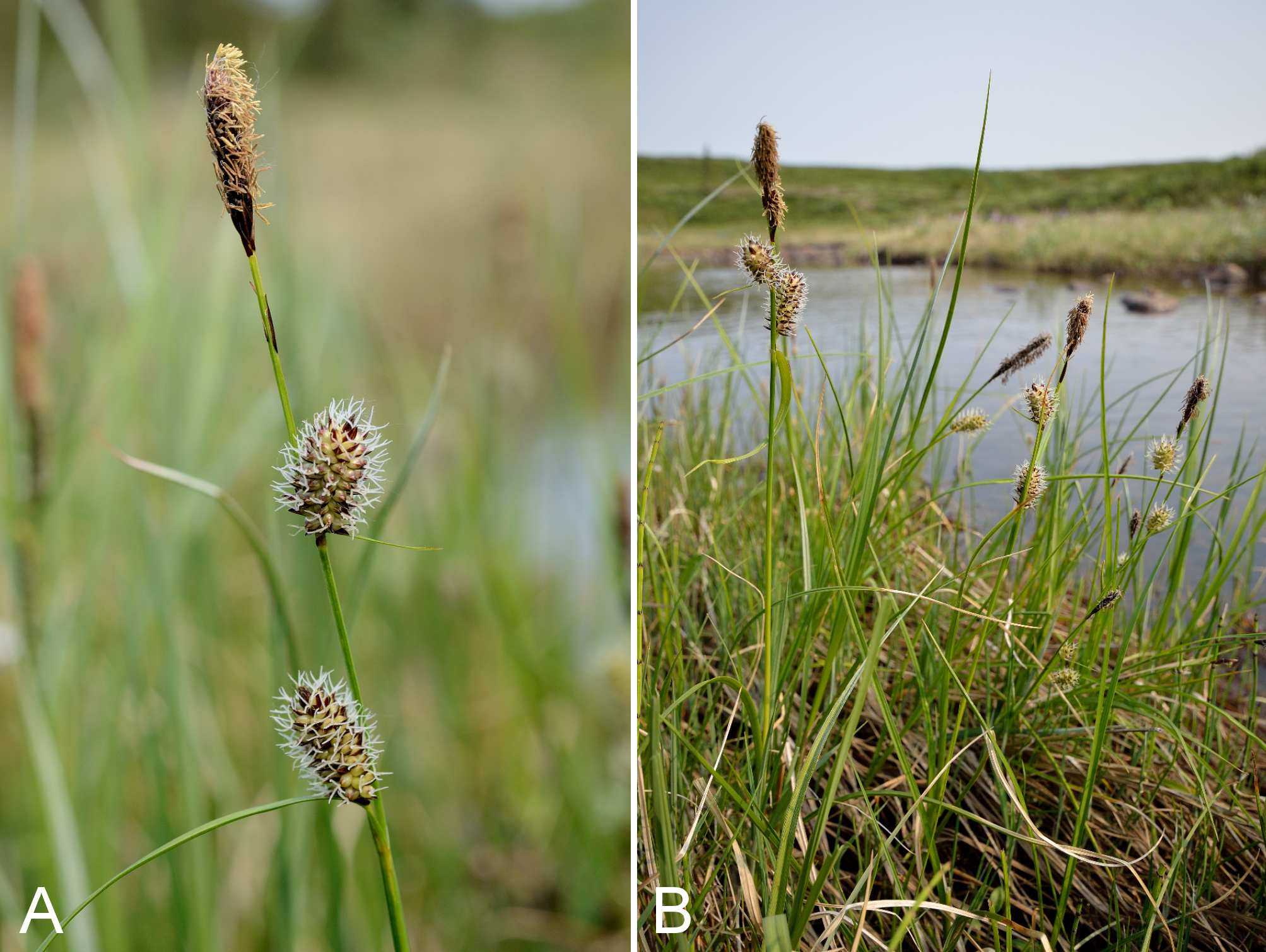

Supplement: Supplemental Information 4 — (A) inflorescence, Saarela et al. 3161. (B) habit, Saarela et al. 3161. Photographs by R. D. Bull. [file peerj-05-2835-s004.png]

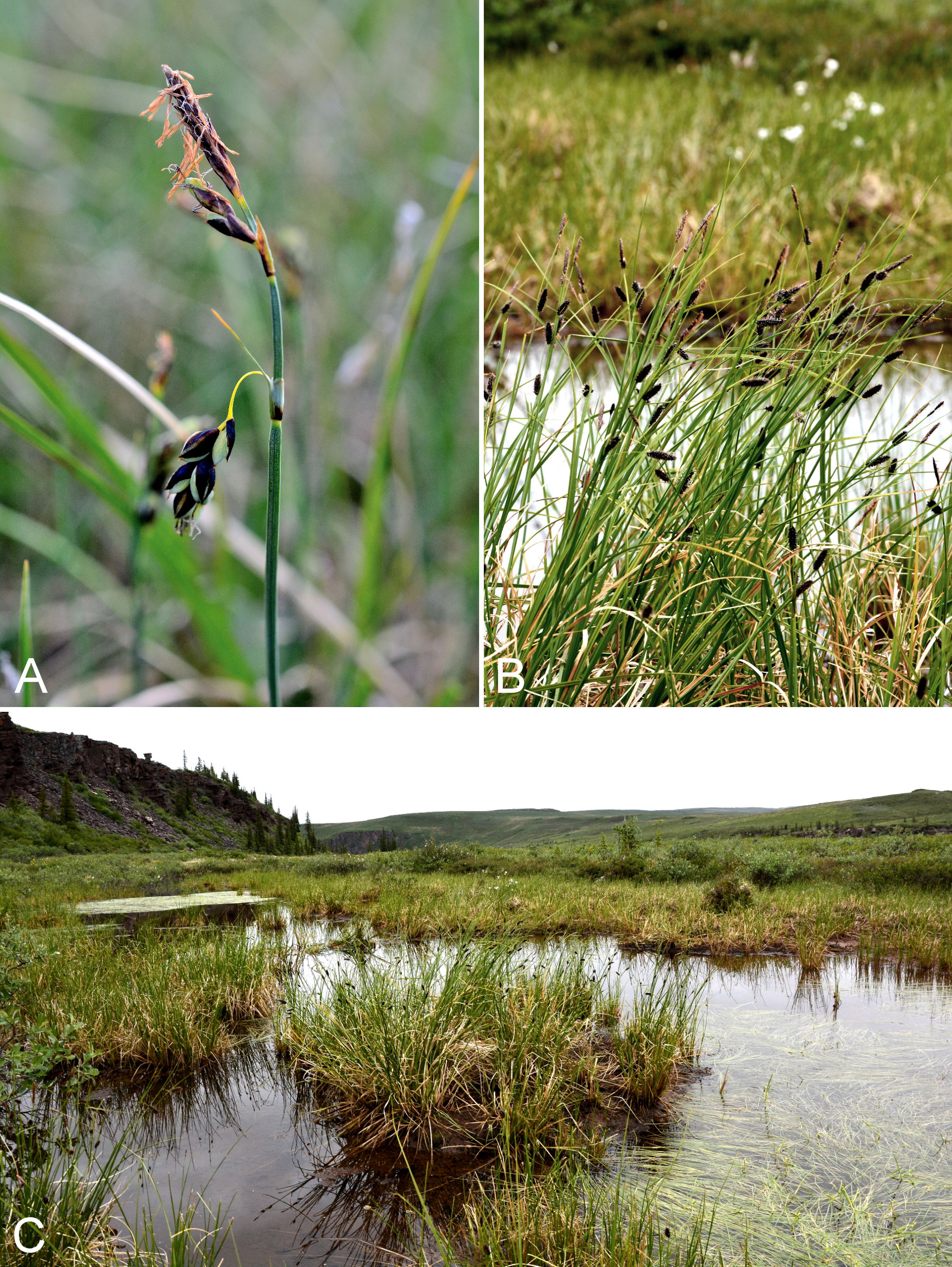

Supplement: Supplemental Information 5 — Carex rariflora: (A) habit, Saarela et al. 4094. Carex saxatilis: (B) habit, Saarela et al. 3607. (C) habitat, Saarela et al. 3607. Photographs by P. C. Sokoloff. [file peerj-05-2835-s005.png]

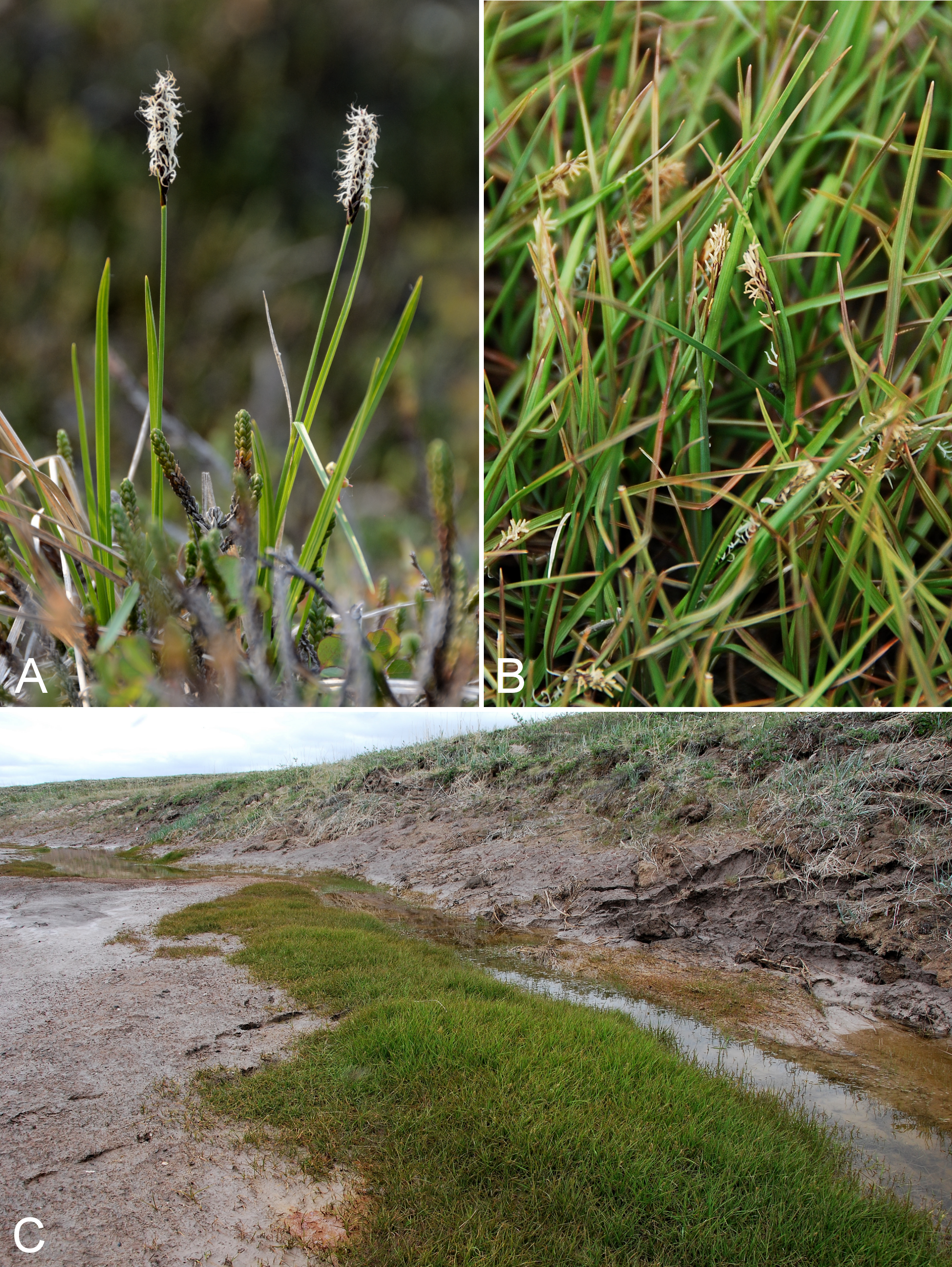

Supplement: Supplemental Information 6 — Carex scirpoidea: (A) inflorescence, vicinity of Bloody Falls, Kugluk (Bloody Falls) Territorial Park, Nunavut, 13 July 2014. Carex subspathacea: (B) habit, Saarela et al. 3670. (C) habitat, Saarela et al. 3670. Photographs by R. D. Bull (A) and J. M. Saarela (B, C). [file peerj-05-2835-s006.png]

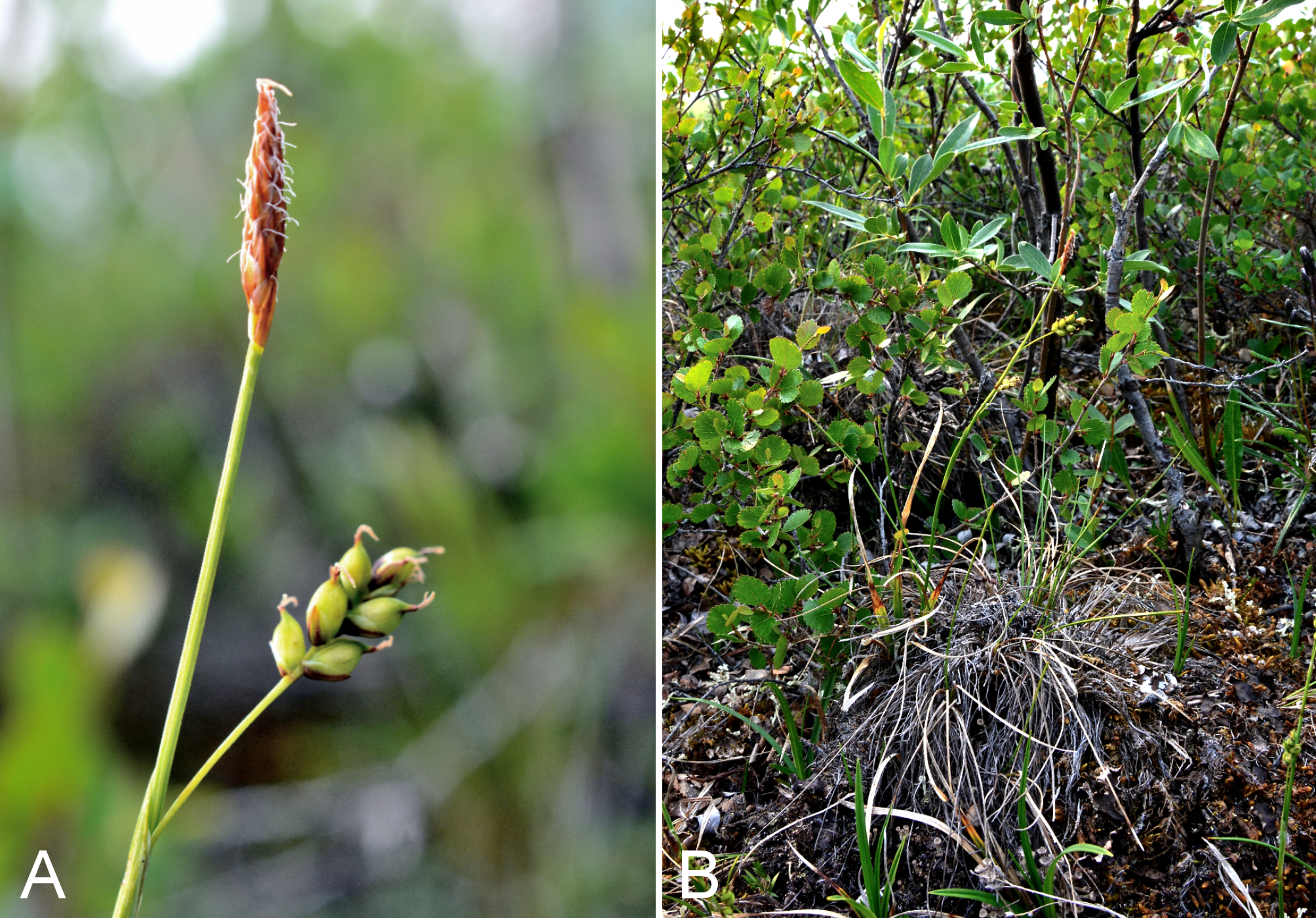

Supplement: Supplemental Information 7 — (A) inflorescence, Saarela et al. 3919. (B) habit, Saarela et al. 3919. Photographs by P. C. Sokoloff. [file peerj-05-2835-s007.png]

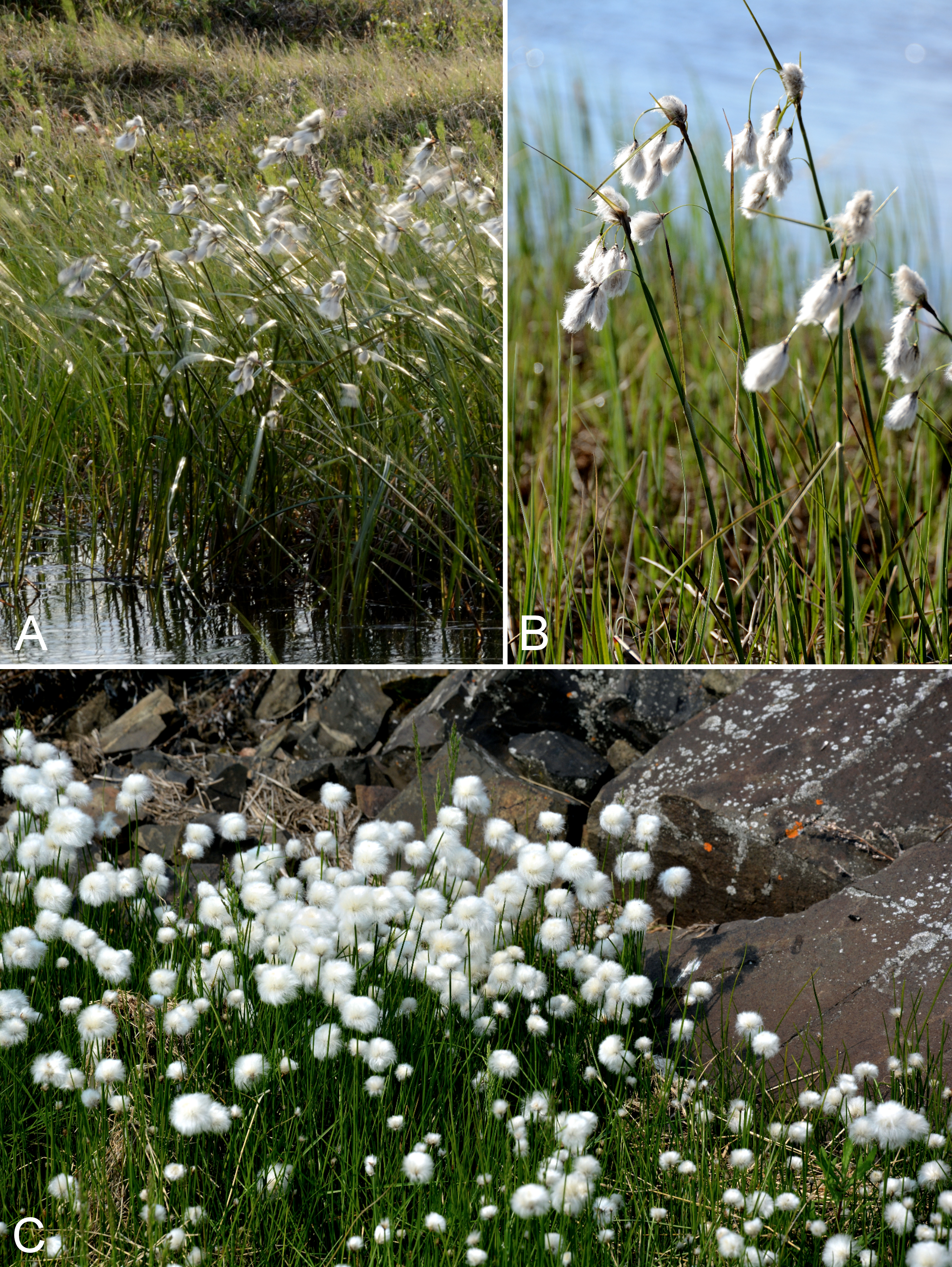

Supplement: Supplemental Information 8 — Eriophorum angustifolium: (A) habitat, Saarela et al. 4003. (B) habit, near Heart Lake, vicinity of Kugluktuk, Nunavut, 23 July 2014. Eriophorum scheuchzeri subsp. arcticum: (C) habit. Photographs by R. D. Bull (A, B) and P. C. Sokoloff (C). [file peerj-05-2835-s008.png]

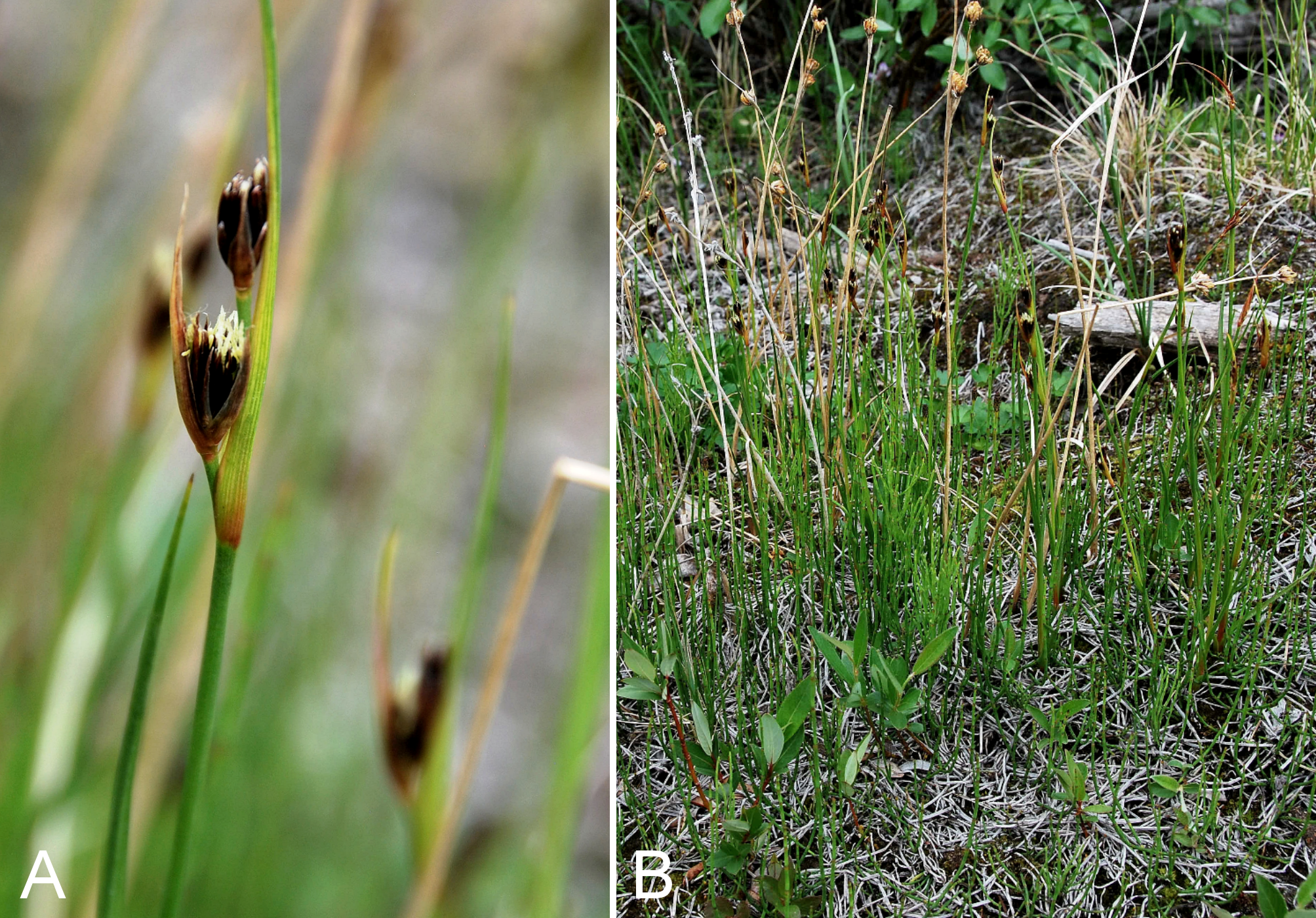

Supplement: Supplemental Information 9 — (A) inflorescence, Saarela et al. 3854. (B) habit, Saarela et al. 3854. Photographs by J. M. Saarela. [file peerj-05-2835-s009.png]

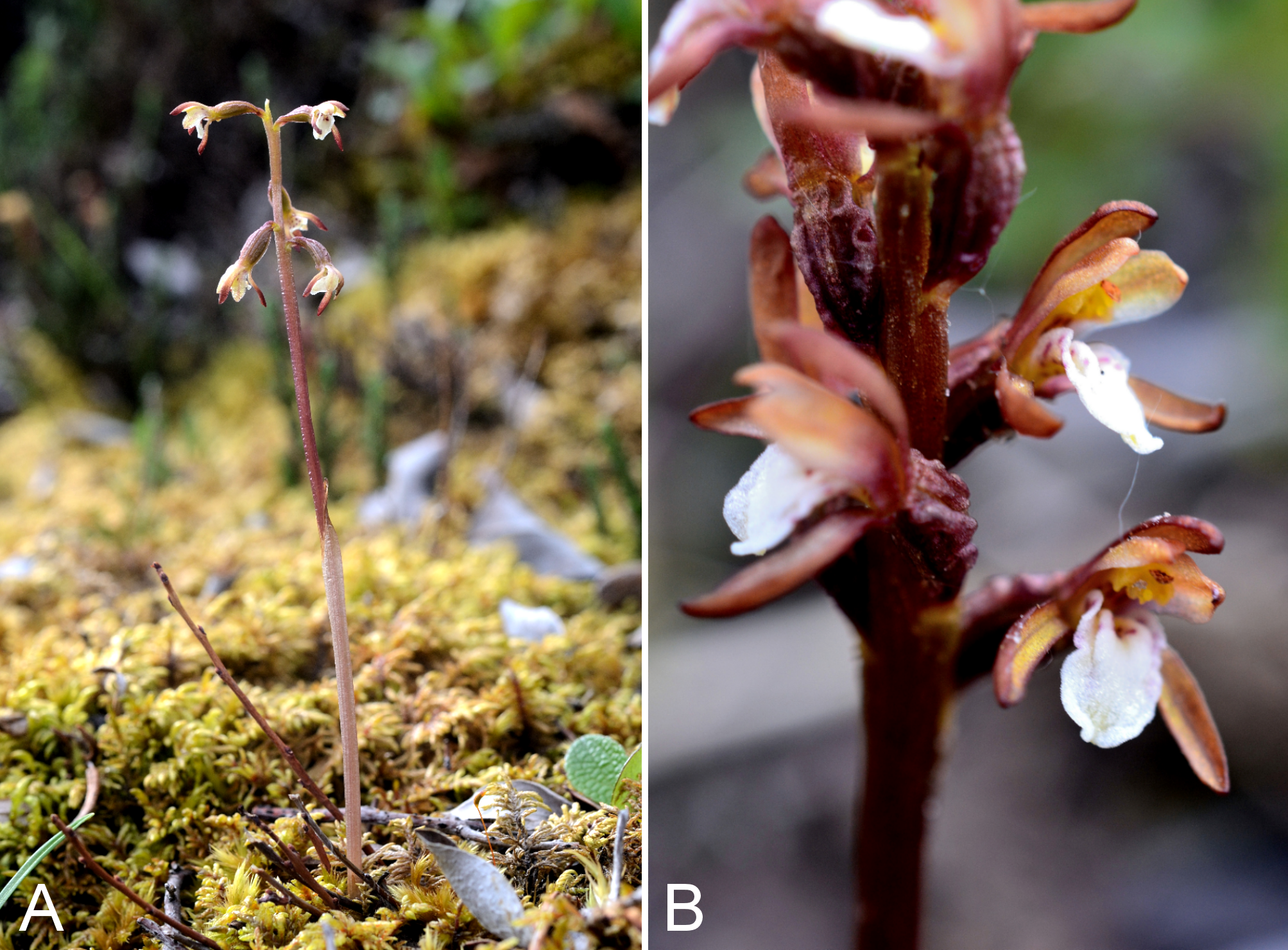

Supplement: Supplemental Information 10 — (A) habit, Saarela et al. 4159. (B) inflorescence, Saarela et al. 3684. Photographs by R. D. Bull (A) and P. C. Sokoloff (B). [file peerj-05-2835-s010.png]

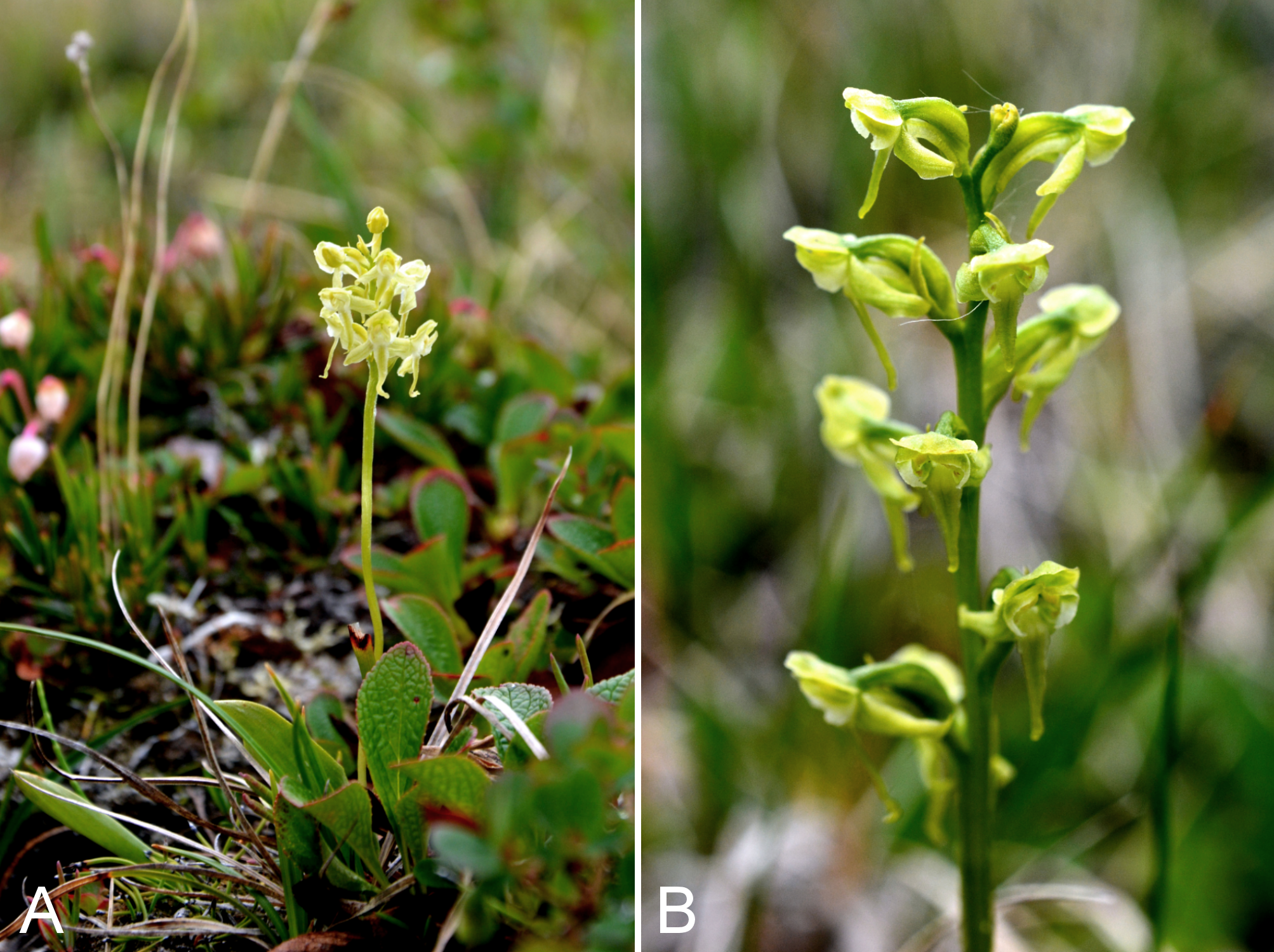

Supplement: Supplemental Information 11 — (A) habit, Saarela et al. 3427. (B) inflorescence, Saarela et al. 3866. Photographs by P. C. Sokoloff. [file peerj-05-2835-s011.png]

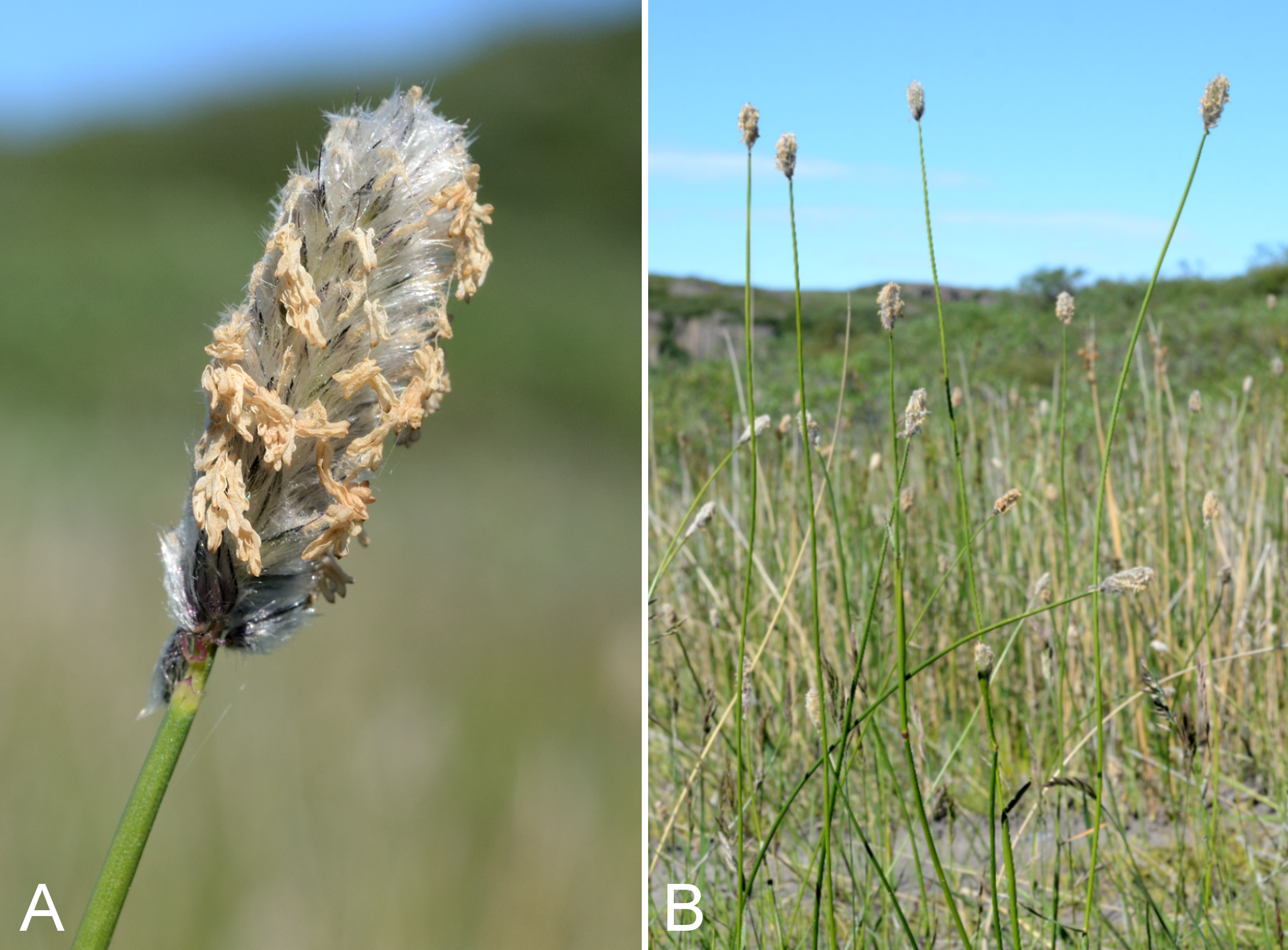

Supplement: Supplemental Information 12 — (A) inflorescence, Saarela et al. 4216. (B) habit, Saarela et al. 4216. Photographs by R. D. Bull. [file peerj-05-2835-s012.png]

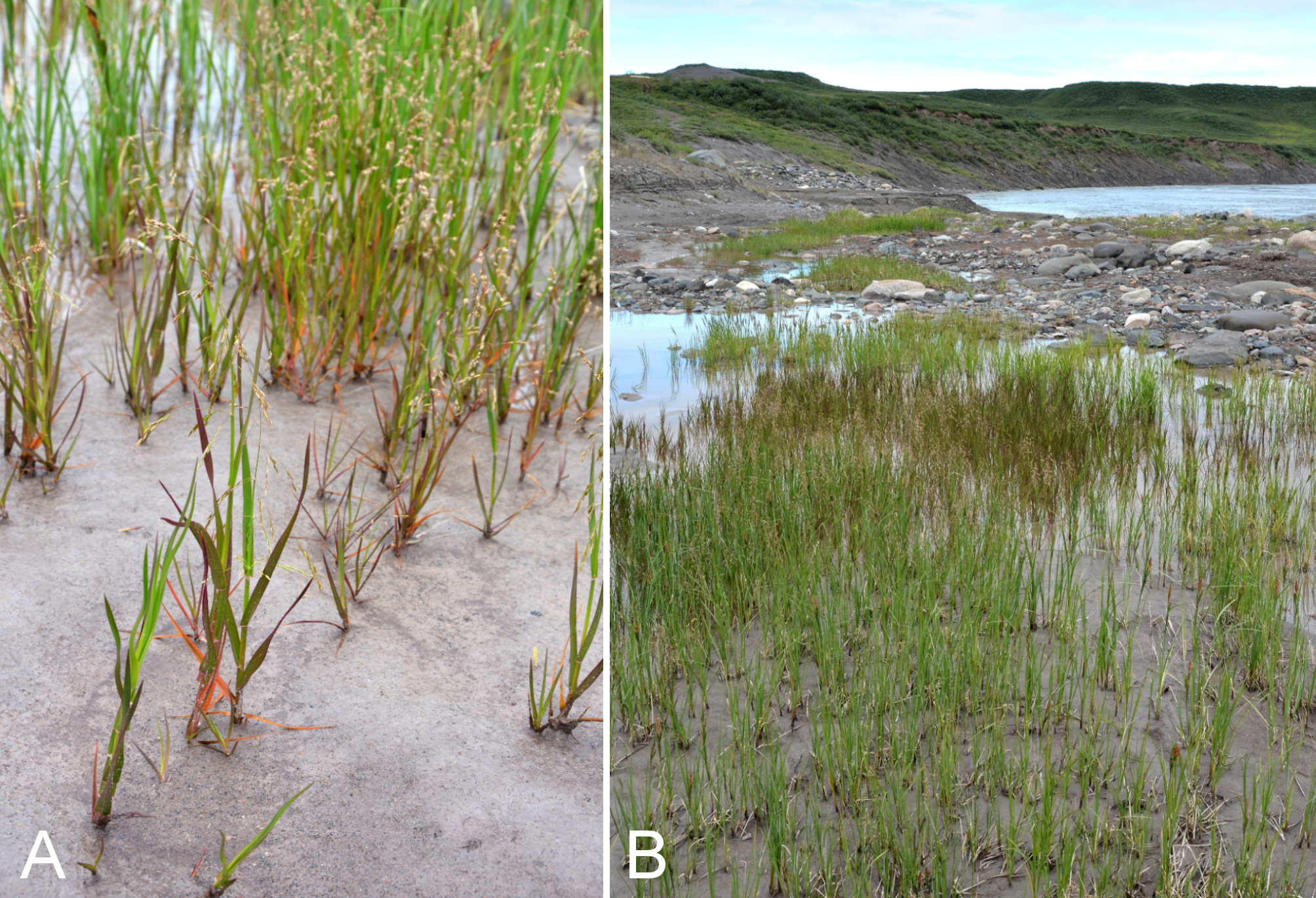

Supplement: Supplemental Information 13 — (A) habit, Saarela et al. 4112. (B) habitat, Saarela et al. 4112. Photographs by P. C. Sokoloff. [file peerj-05-2835-s013.png]

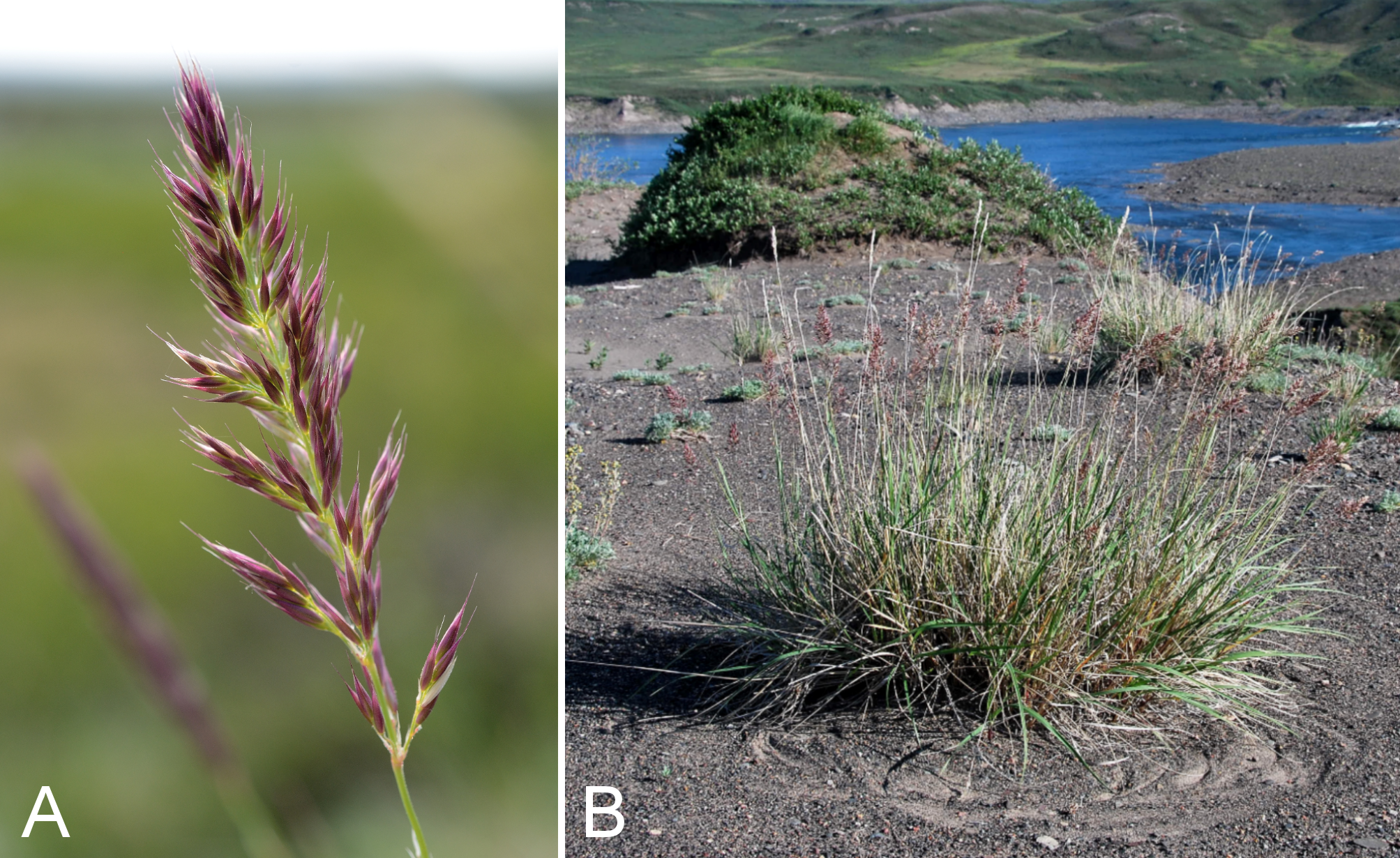

Supplement: Supplemental Information 14 — (A) inflorescence, vicinity of Fockler Creek, Nunavut, 4 July 2014. (B) habit, Kugluk (Bloody Falls) Territorial Park, Nunavut, 18 July 2014. Photographs by R. D. Bull (A) and J. M. Saarela (B). [file peerj-05-2835-s014.png]

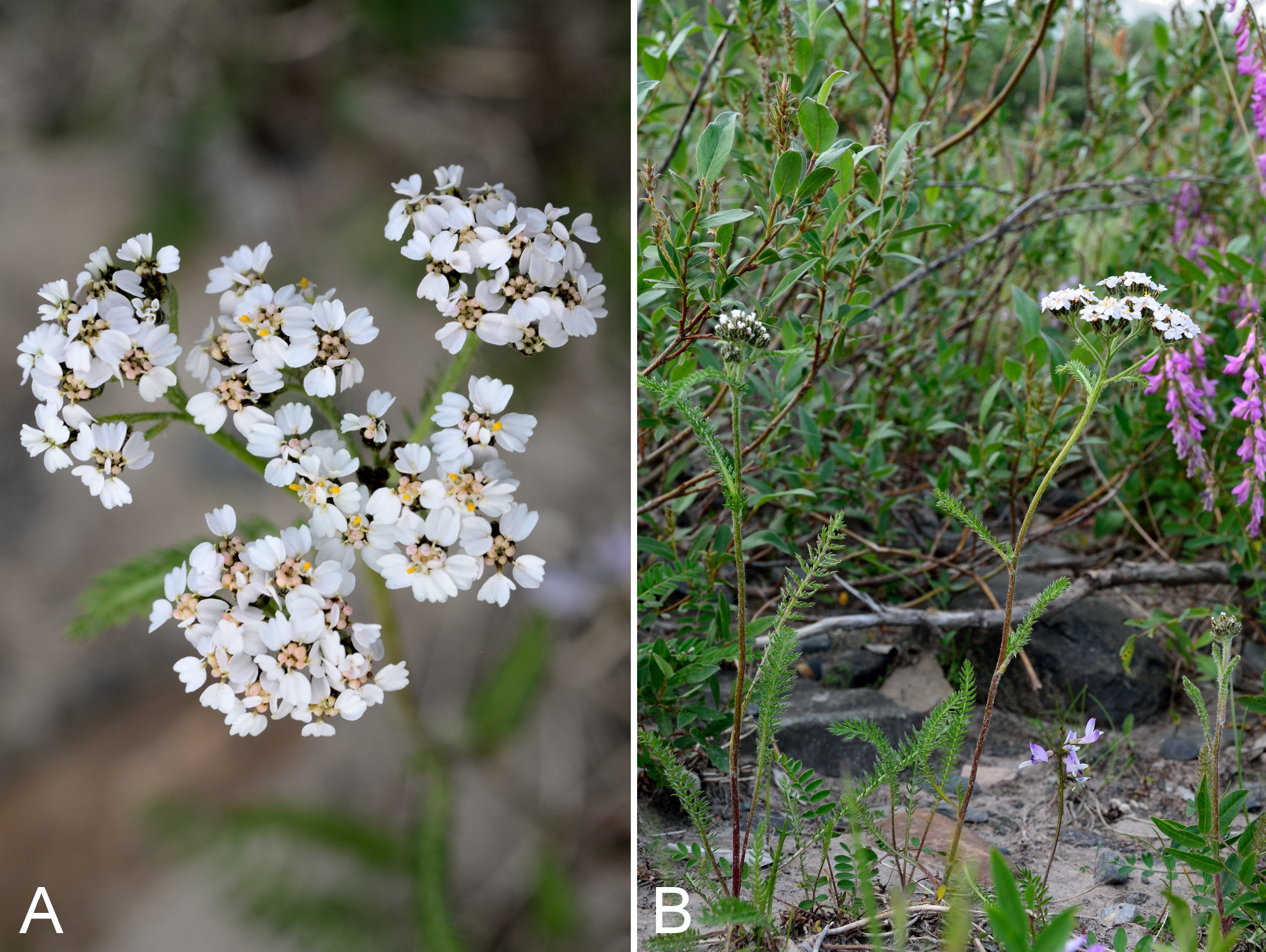

Supplement: Supplemental Information 15 — (A) capitulum, Saarela et al. 3959. (B) habit, Saarela et al. 3959. Photographs by R. D. Bull. [file peerj-05-2835-s015.png]

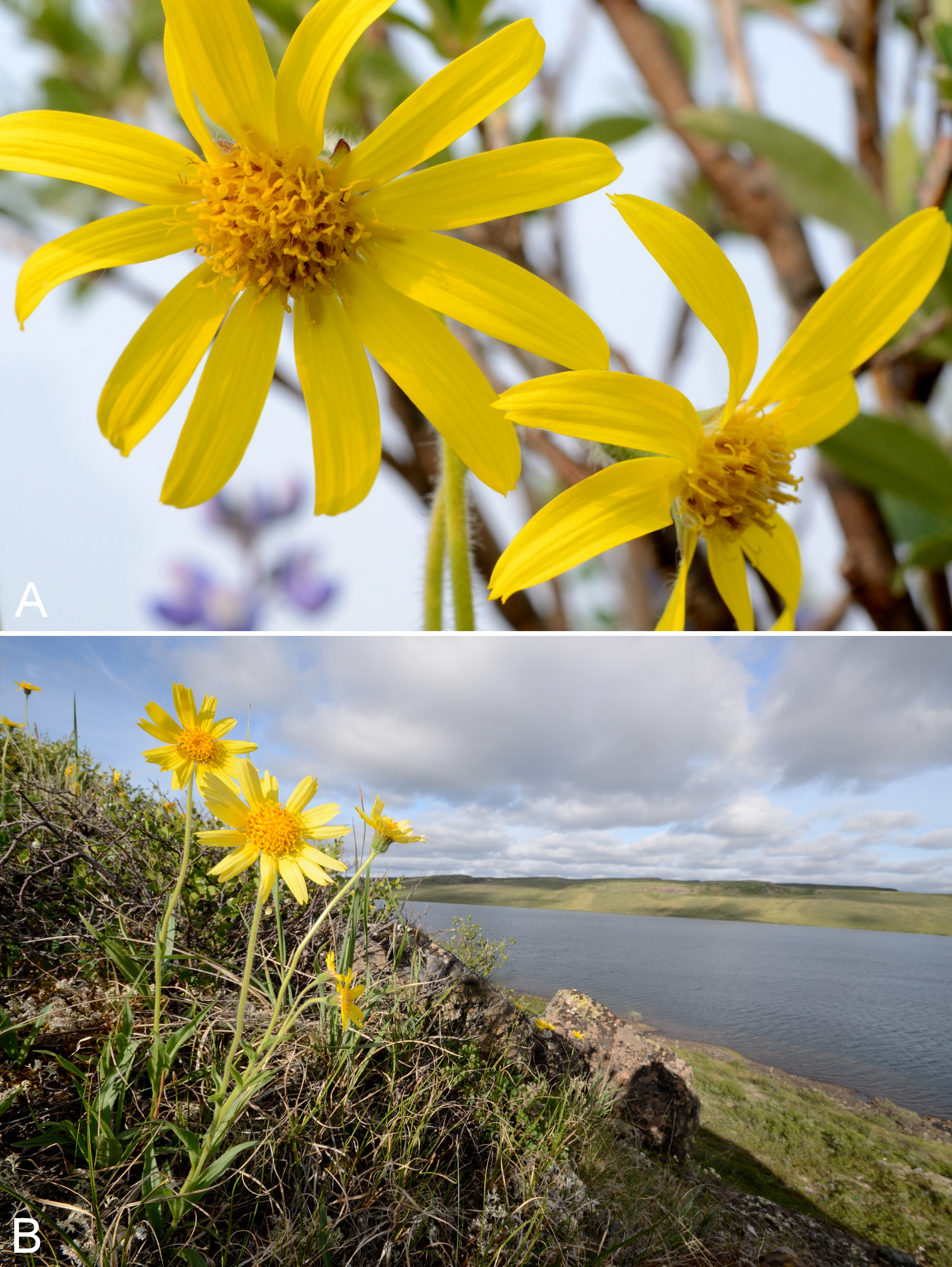

Supplement: Supplemental Information 16 — (A) capitulum, vicinity of Fockler Creek, Nunavut, 4 July 2014. (B) habit, vicinity of Tundra Lake, Nunavut, 5 July 2014. Photographs by R. D. Bull. [file peerj-05-2835-s016.png]

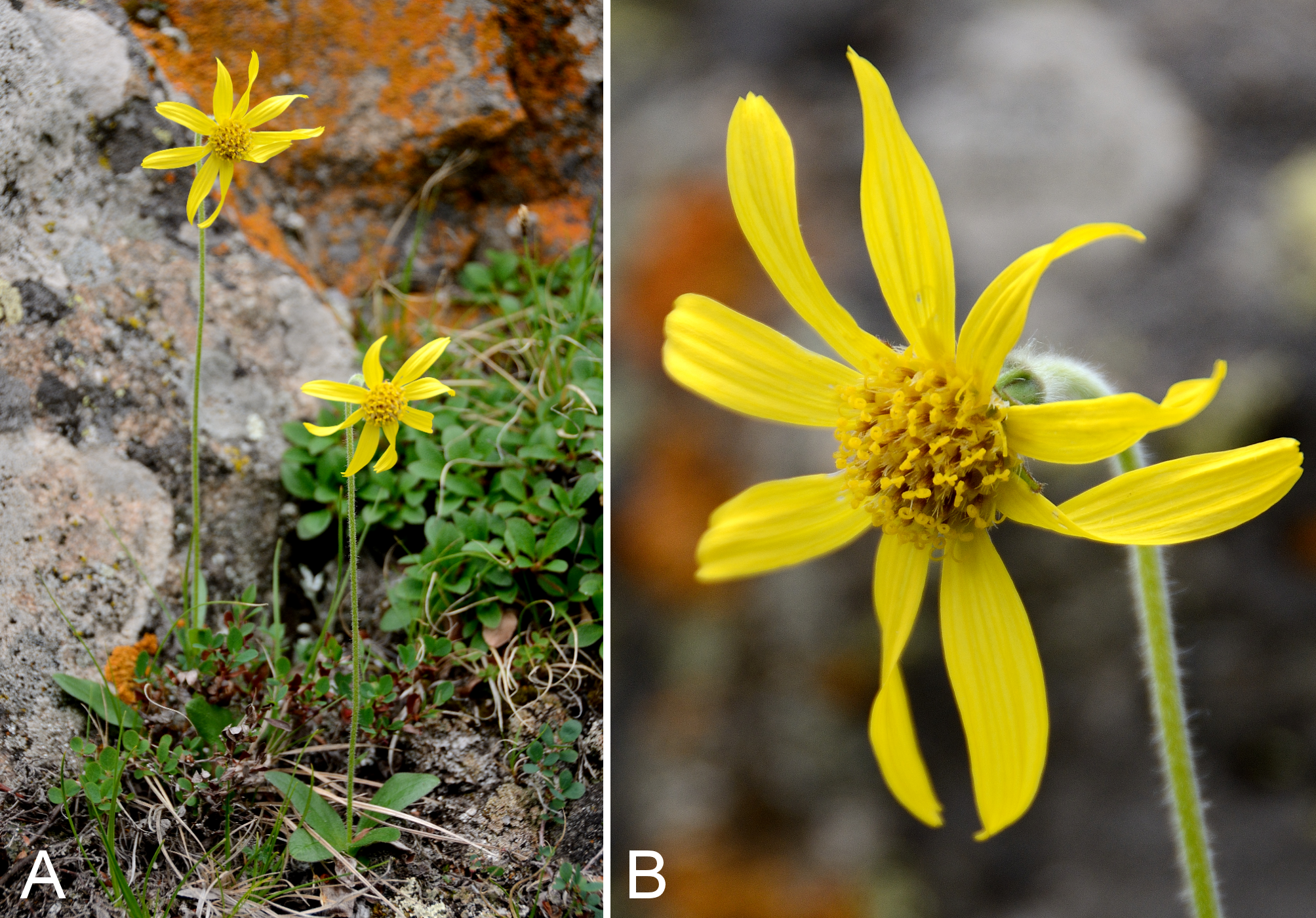

Supplement: Supplemental Information 17 — (A) habit, Saarela et al.4371. (B) capitulum, Saarela et al. 4371. Photographs by R. D. Bull. [file peerj-05-2835-s017.png]

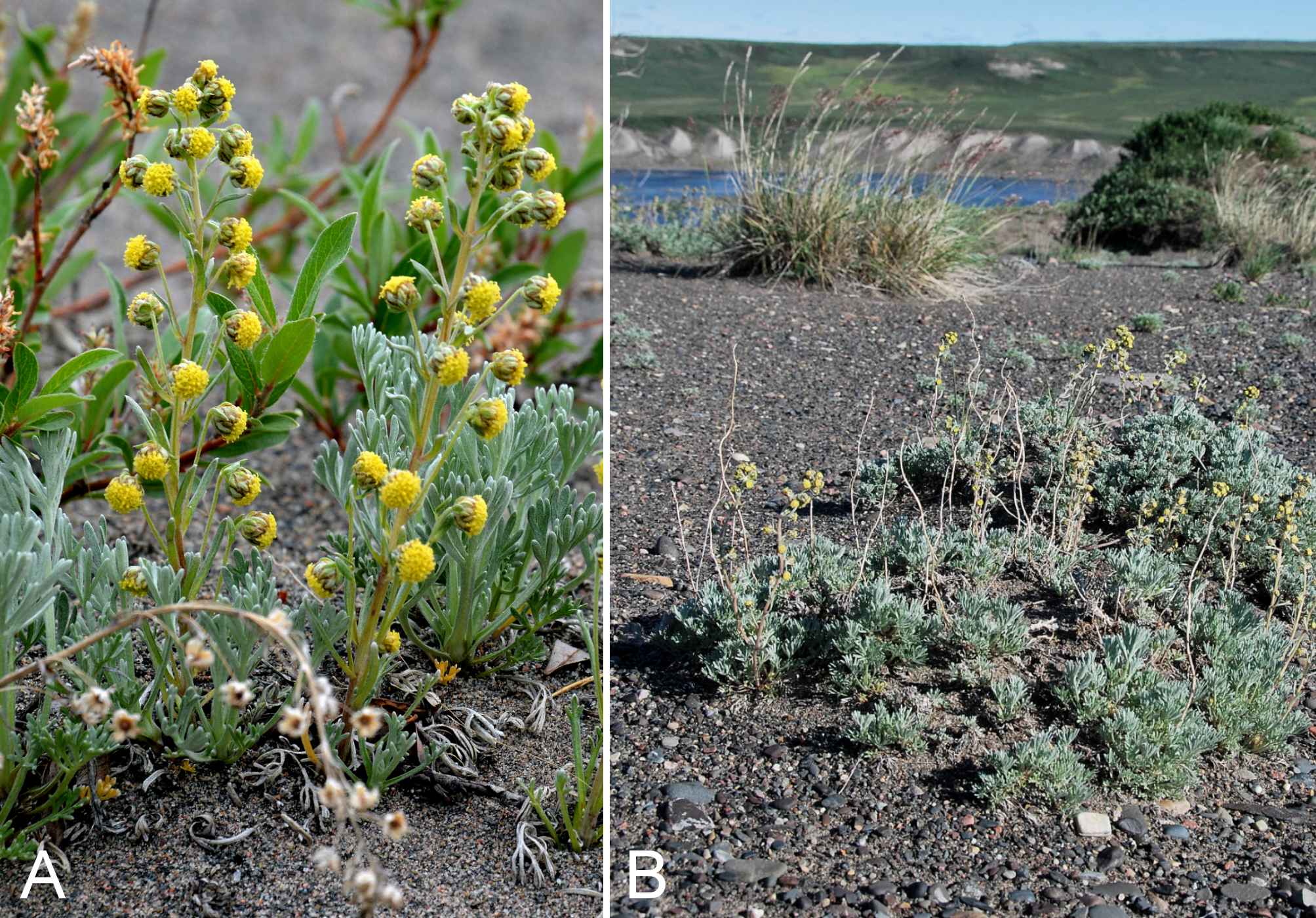

Supplement: Supplemental Information 18 — (A) habit, Saarela et al. 4020. (B) habitat, Saarela et al. 4020. Photographs by J. M. Saarela (A) and P. C. Sokoloff (B). [file peerj-05-2835-s018.png]

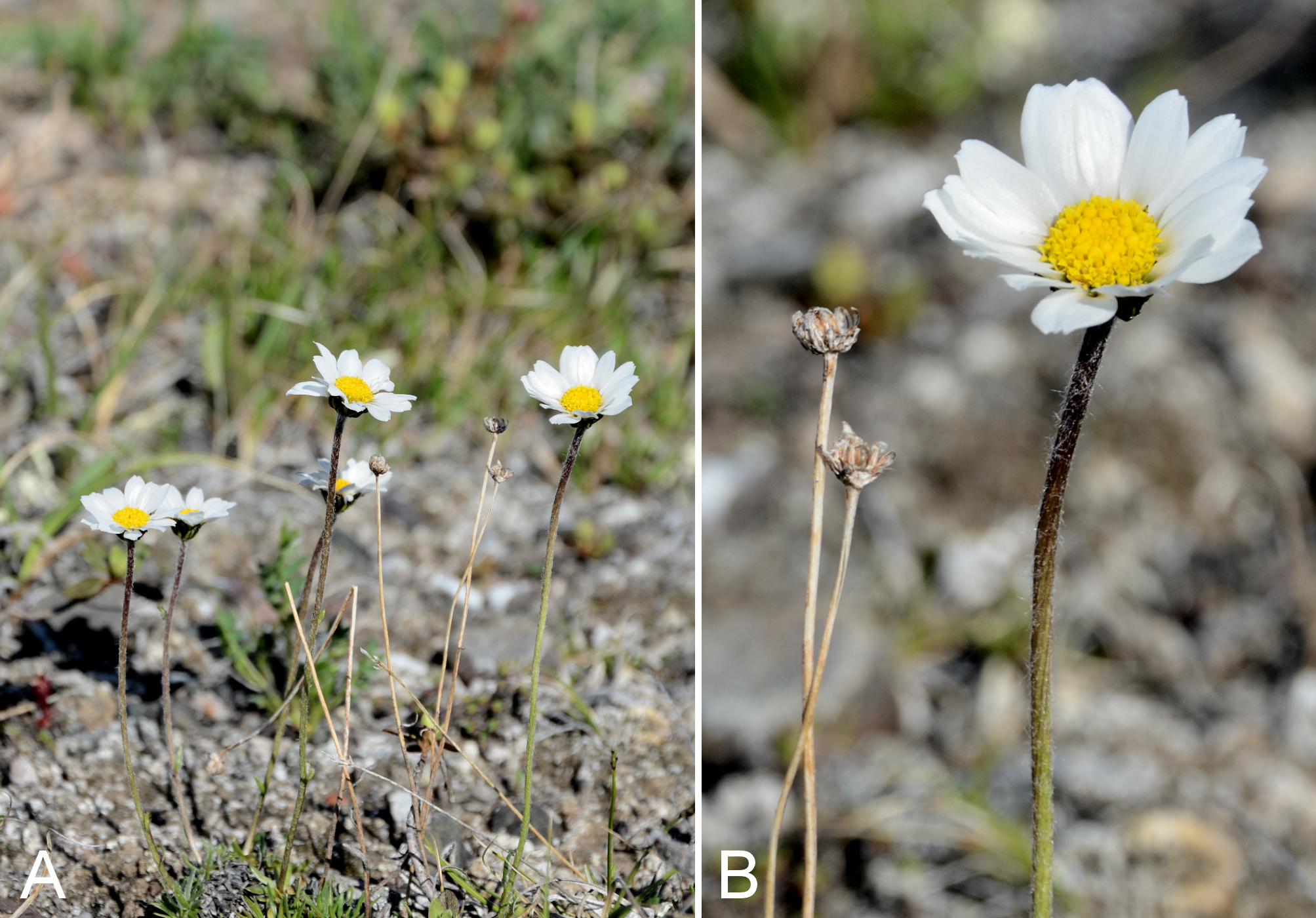

Supplement: Supplemental Information 19 — (A) habit, vicinity of Fockler Creek, Nunavut, 6 July 2014. (B) capitulum, vicinity of Fockler Creek, Nunavut, 6 July 2014. Photographs by R. D. Bull. [file peerj-05-2835-s019.png]

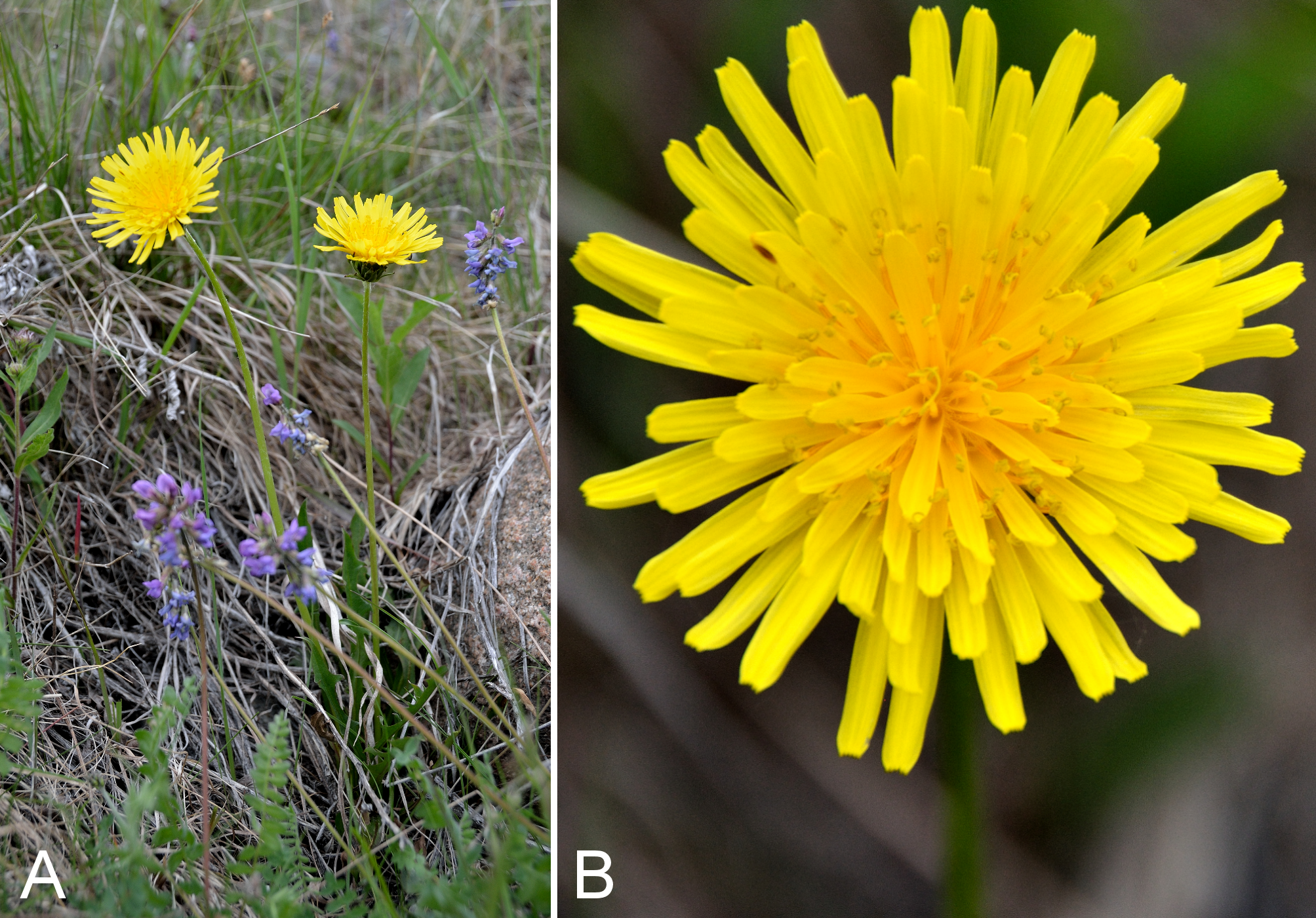

Supplement: Supplemental Information 20 — (A) habit, vicinity of lower Coppermine River, Nunavut, 8 July 2014. (B) capitulum, vicinity of lower Coppermine River, Nunavut, 8 July 2014. Photographs by R. D. Bull. [file peerj-05-2835-s020.png]

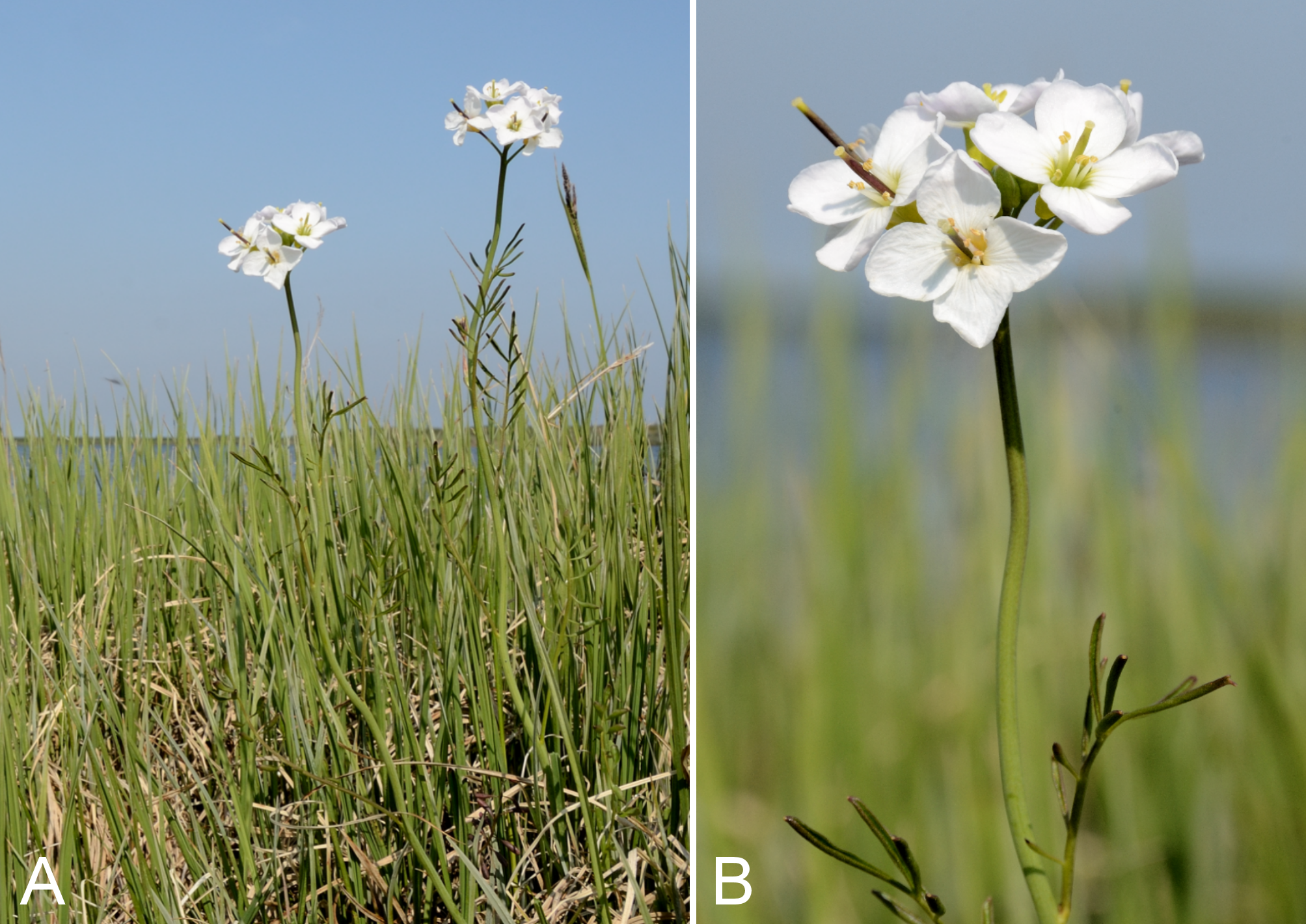

Supplement: Supplemental Information 21 — (A) habit, Saarela et al. 4319. (B) inflorescence, Saarela et al. 4319. Photographs by R. D. Bull. [file peerj-05-2835-s021.png]

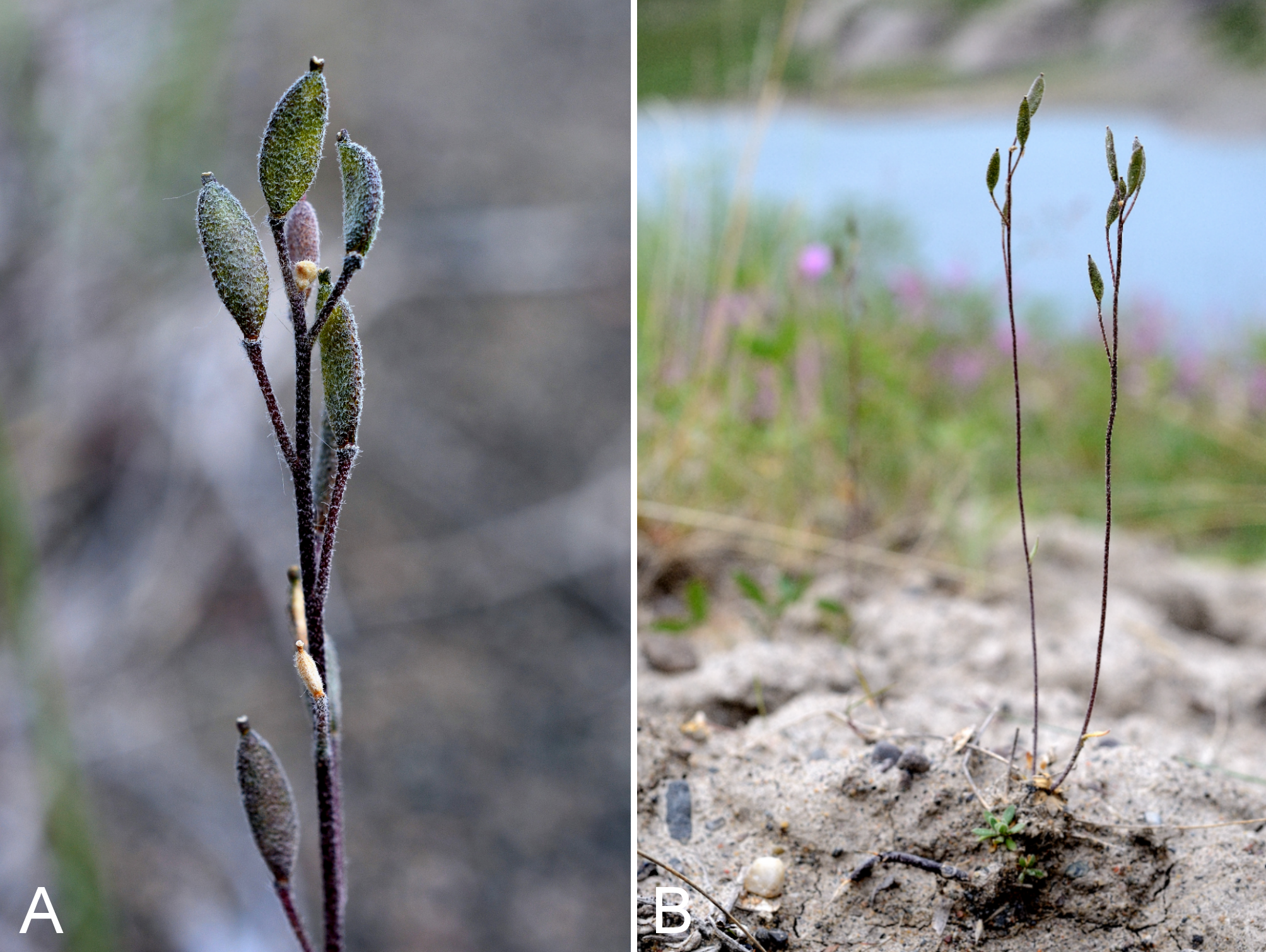

Supplement: Supplemental Information 22 — (A) fruits, Saarela et al. 4180. (B) habit, Saarela et al. 4199. Photographs by P. C. Sokoloff (A) and R. D. Bull (B). [file peerj-05-2835-s022.png]

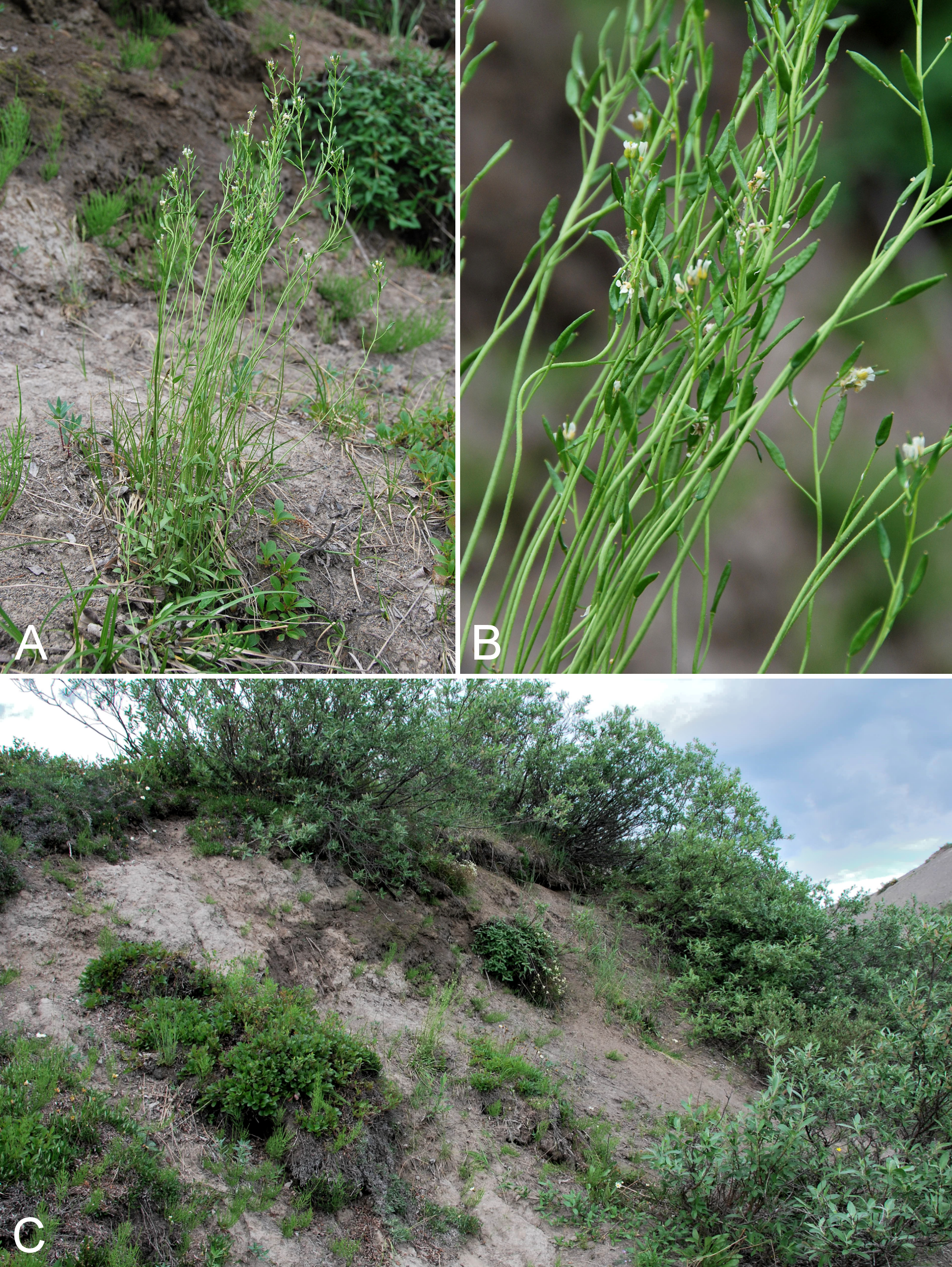

Supplement: Supplemental Information 23 — (A) habit, Saarela et al. 4018. (B) fruits, Saarela et al. 4018. (C) habitat, Saarela et al. 4018. Photographs by J. M. Saarela. [file peerj-05-2835-s023.png]

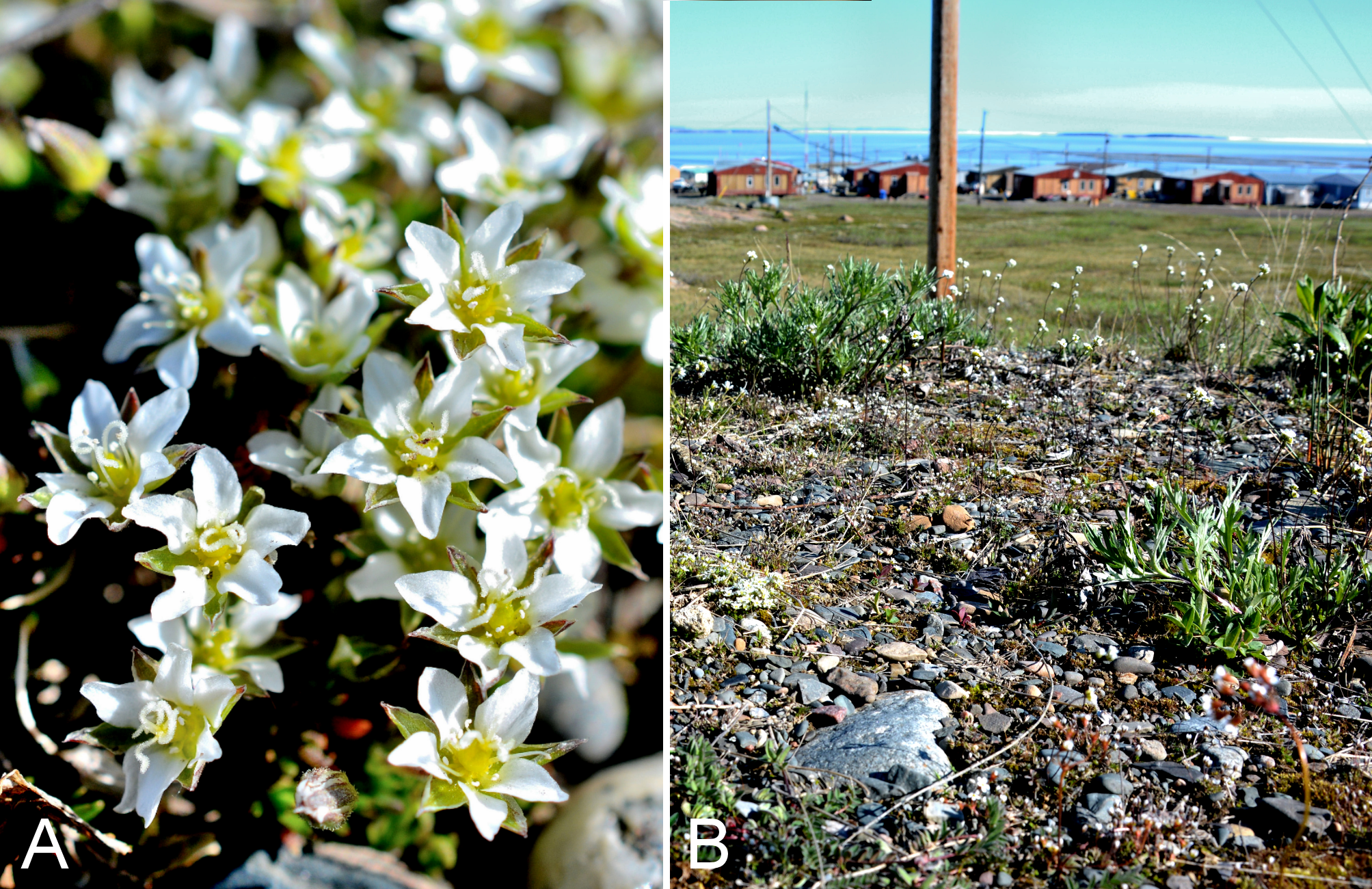

Supplement: Supplemental Information 24 — (A) inflorescence, Saarela et al. 3096. (B) habitat, Saarela et al. 3096. Photographs by P. C. Sokoloff. [file peerj-05-2835-s024.png]

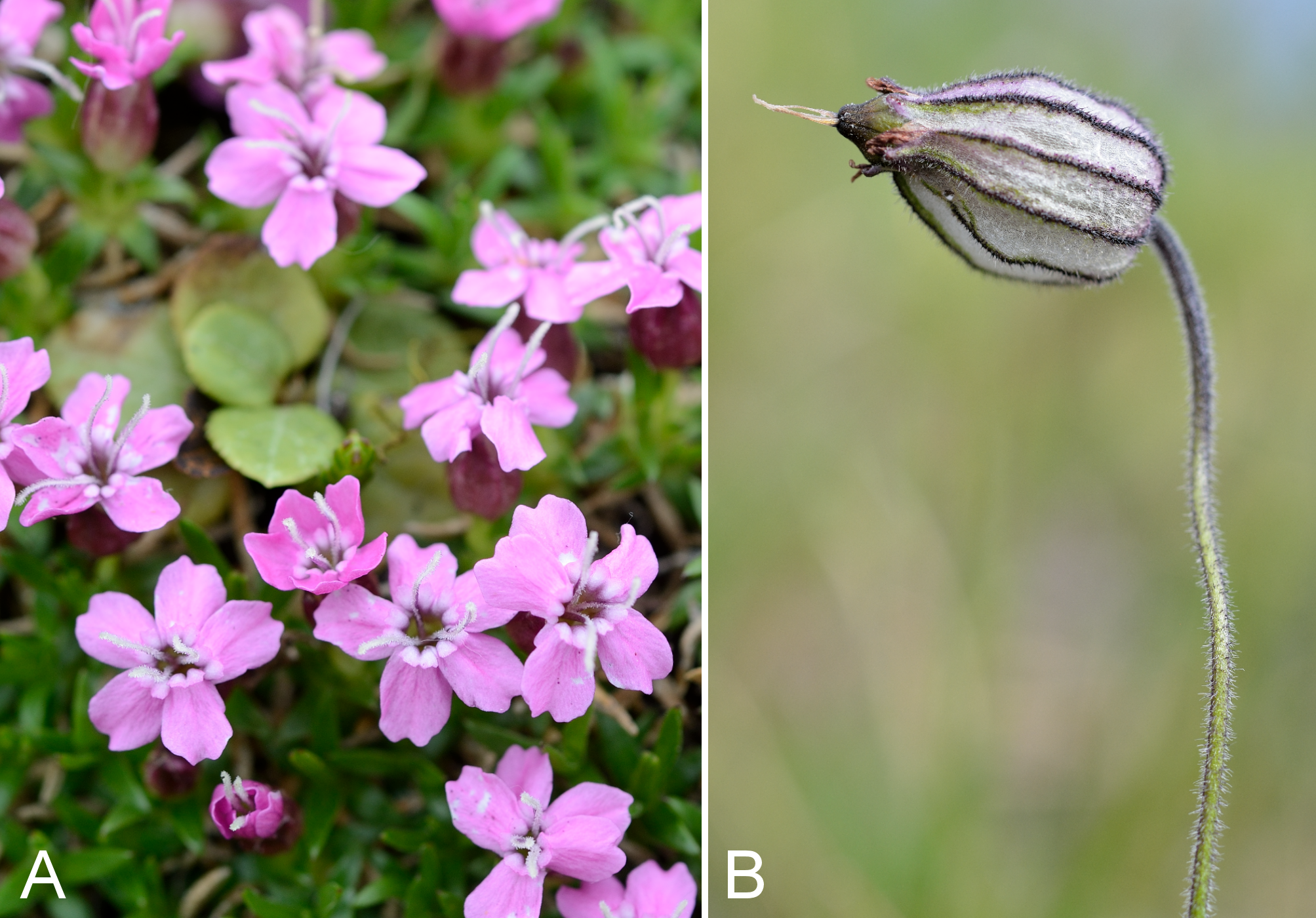

Supplement: Supplemental Information 25 — Silene acaulis: (A) inflorescences, Kugluk (Bloody Falls) Territorial Park, Nunavut, 13 July 2014. Silene uralensis subsp. uralensis: (B) inflorescence, Saarela et al. 4050. Photographs by R. D. Bull. [file peerj-05-2835-s025.png]

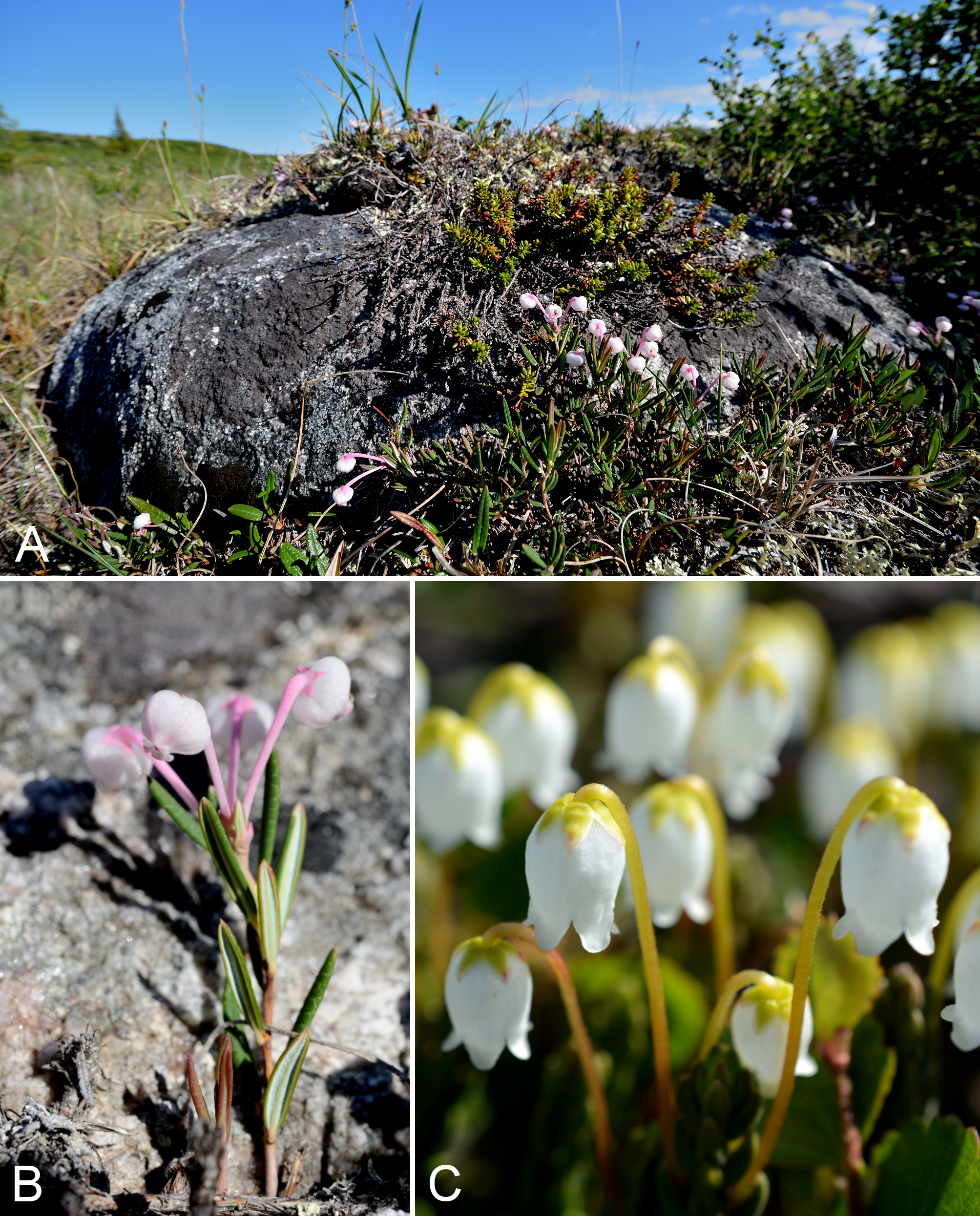

Supplement: Supplemental Information 26 — Andromeda polifolia: (A) habitat, Saarela et al. 3208. (B) habit, Saarela et al. 3208. Cassiope tetragona subsp. tetragona: (C) inflorescence, vicinity of Kugluktuk Airport, 30 June 2014. Photographs by R. D. Bull (A, B) and P. C. Sokoloff (C). [file peerj-05-2835-s026.png]

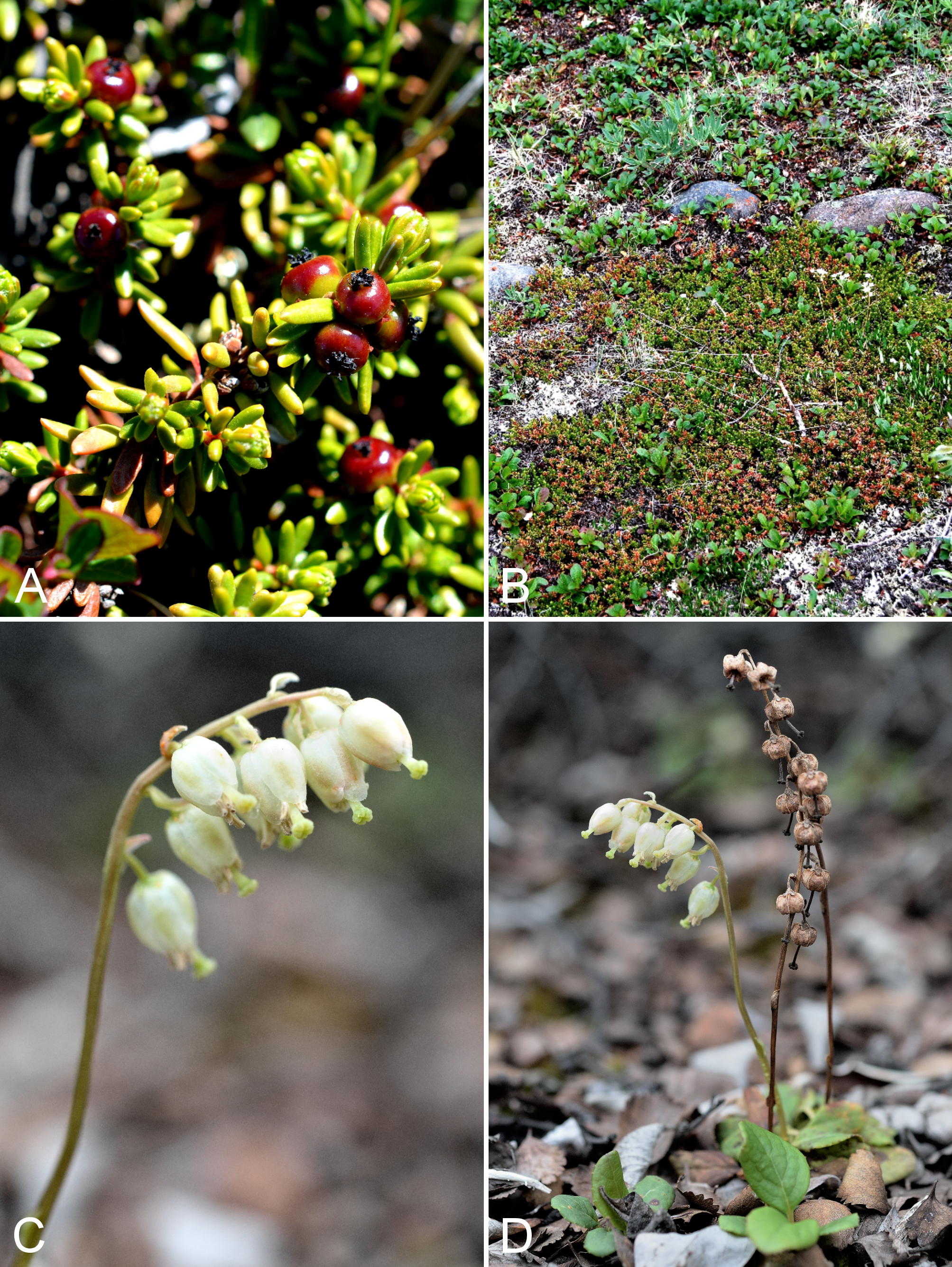

Supplement: Supplemental Information 27 — Empetrum nigrum: (A) young fruits, vicinity of Kugluktuk Airport, 30 June 2014. (B) habitat, Coppermine Mountains, 9 July 2014. Orthilia secunda subsp. obtusata: (C) inflorescence, Saarela et al. 4139. (D) habit, Saarela et al. 4139. P. C. Sokoloff (A), J. M. Saarela (B) and R. D. Bull (C, D). [file peerj-05-2835-s027.png]

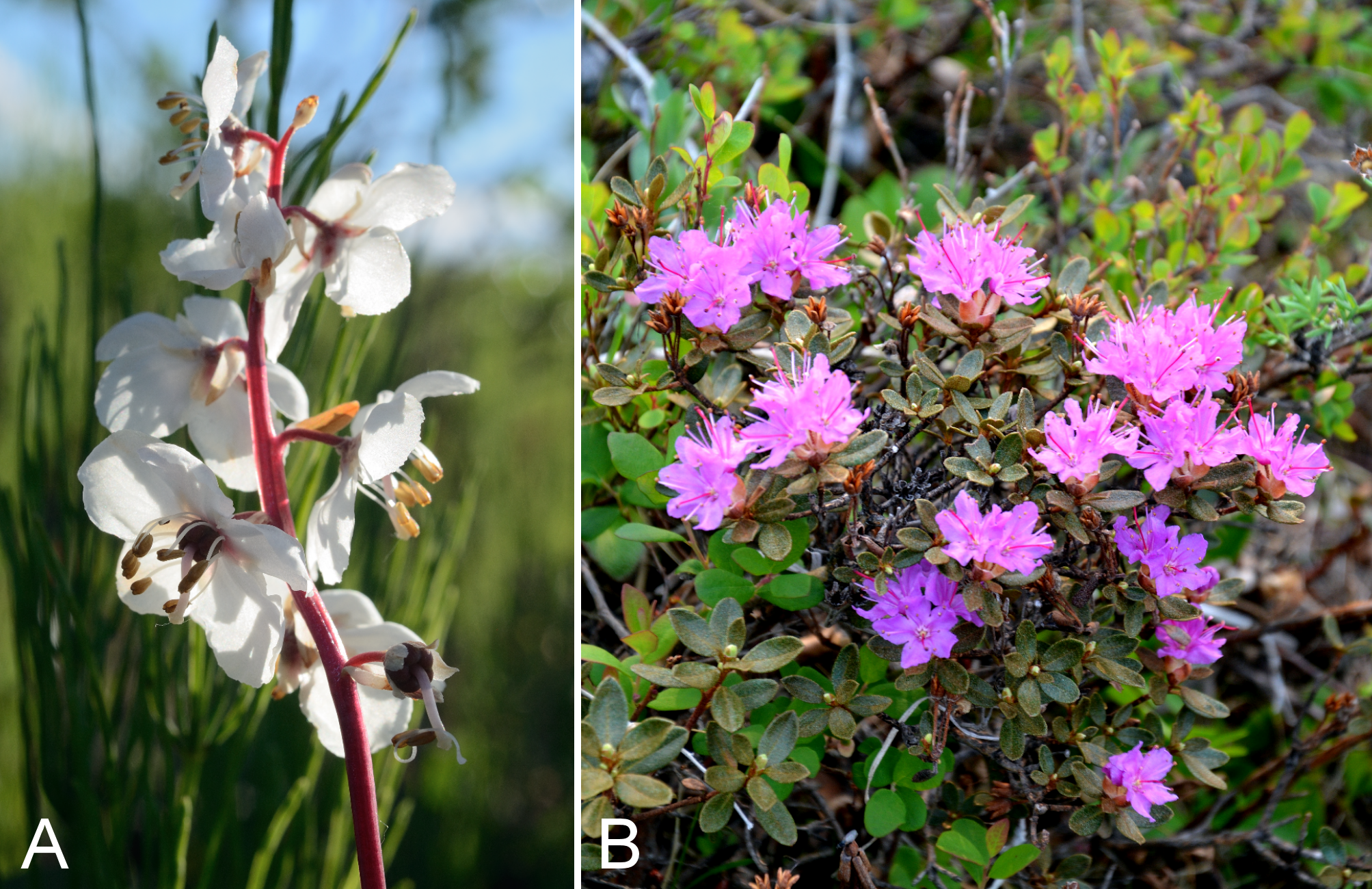

Supplement: Supplemental Information 28 — Pyrola grandiflora: (A) inflorescence, Kugluk (Bloody Falls) Territorial Park, Nunavut, 16 July 2014. Rhododendron lapponicum: (B) habit, Saarela et al. 3245. Photographs by R. D. Bull (A) and P. C. Sokoloff (B). [file peerj-05-2835-s028.png]

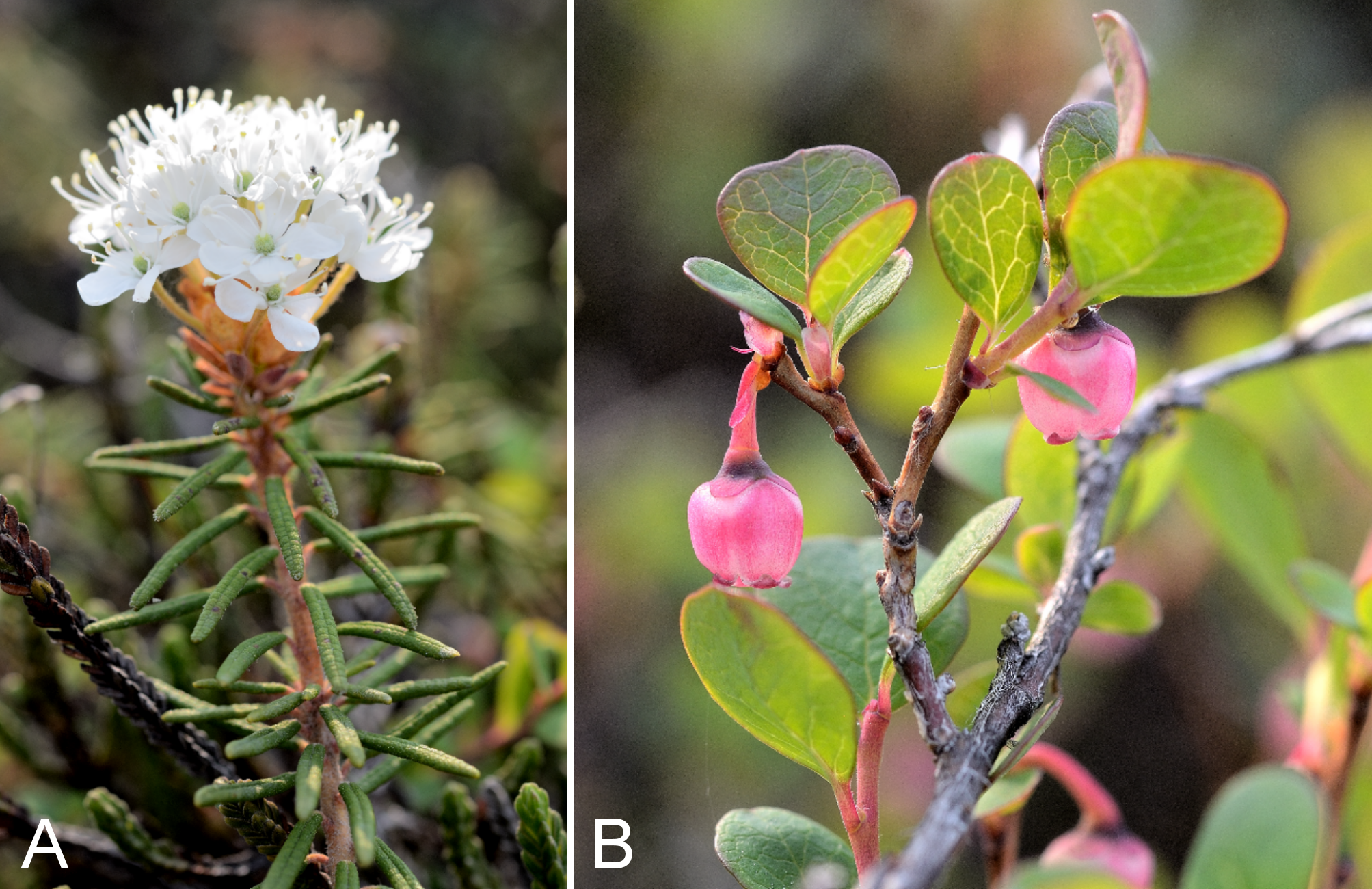

Supplement: Supplemental Information 29 — Rhododendron tomentosum subsp. decumbens: (A) inflorescence, Kugluk (Bloody Falls) Territorial Park, Nunavut, 13 July 2014. Vaccinium uliginosum: (B) inflorescence, Kugluk (Bloody Falls) Territorial Park, Nunavut, 13 July 2014. Photographs by R. D. Bull. [file peerj-05-2835-s029.png]

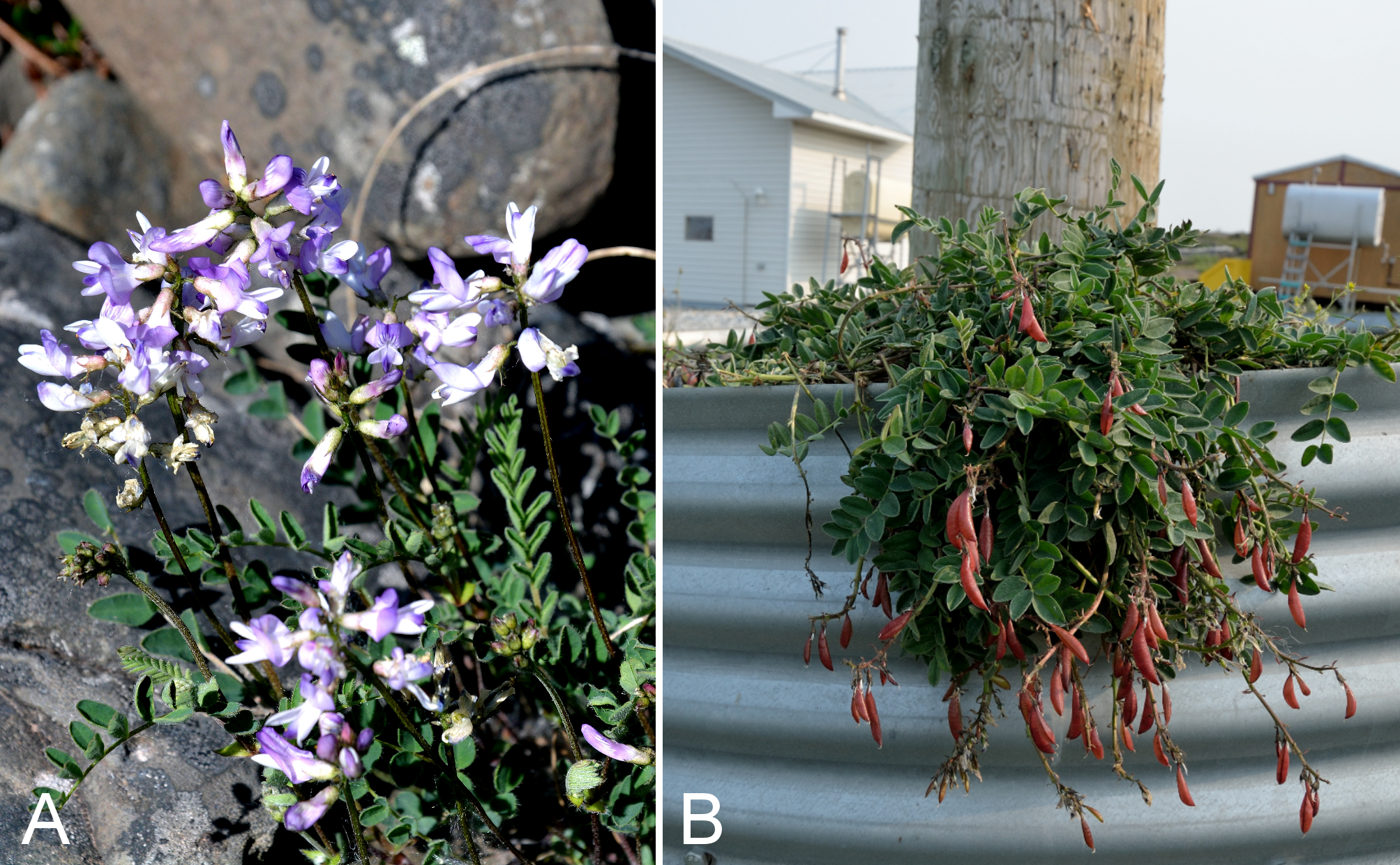

Supplement: Supplemental Information 30 — Astragalus alpinus: (A) habit, Saarela et al. 3159. Astragalus richardsonii: (B) habit, Kugluktuk, Nunavut, 24 July 2014. Photographs by P. C. Sokoloff (A) and R. D. Bull (B). [file peerj-05-2835-s030.png]

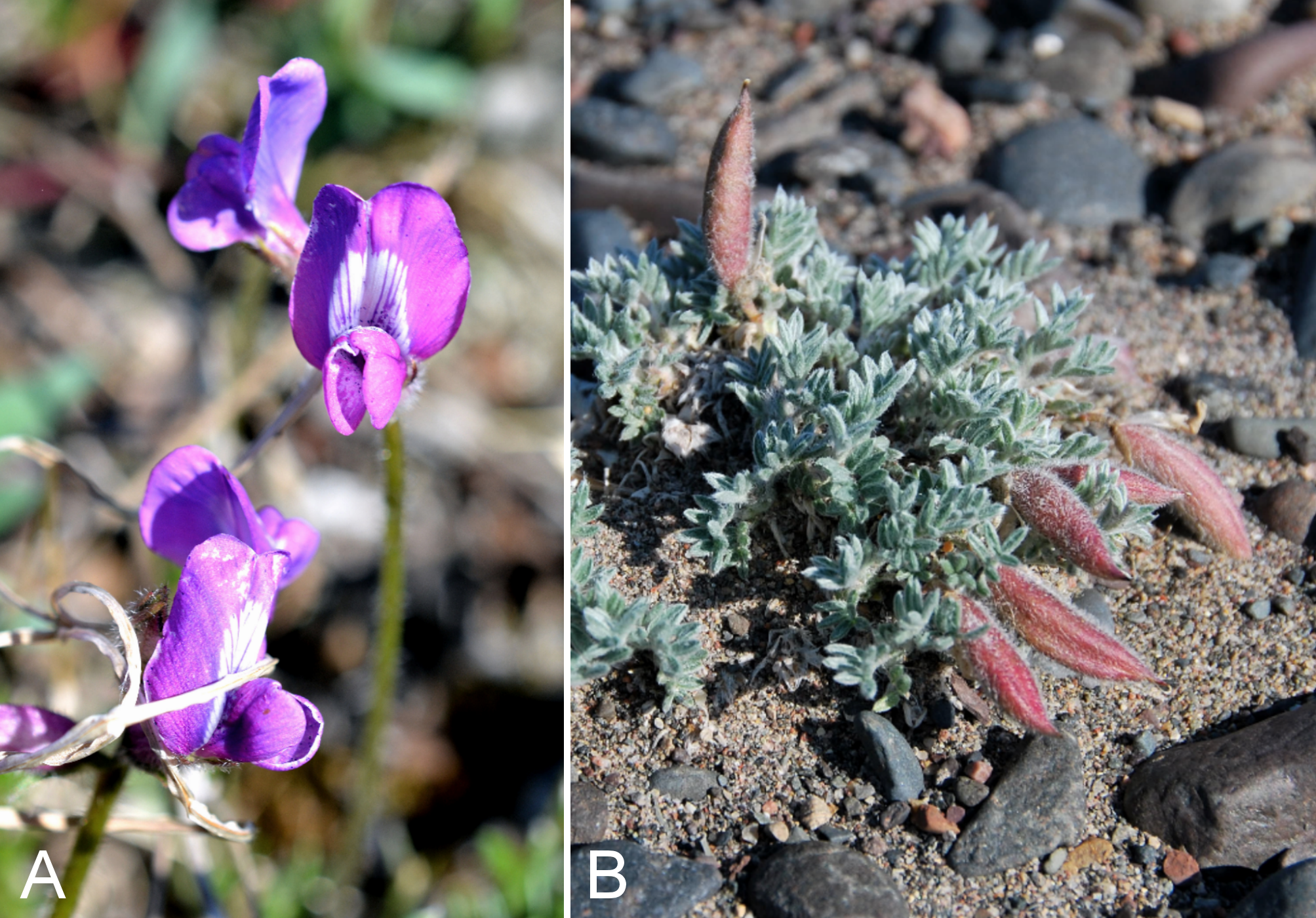

Supplement: Supplemental Information 31 — Oxytropis arctica: (A) inflorescence, Saarela et al. 3177. Oxytropis arctobia: (B) habit, Saarela et al. 4178. Photographs by P. C. Sokoloff (A) and J. M. Saarela (B). [file peerj-05-2835-s031.png]

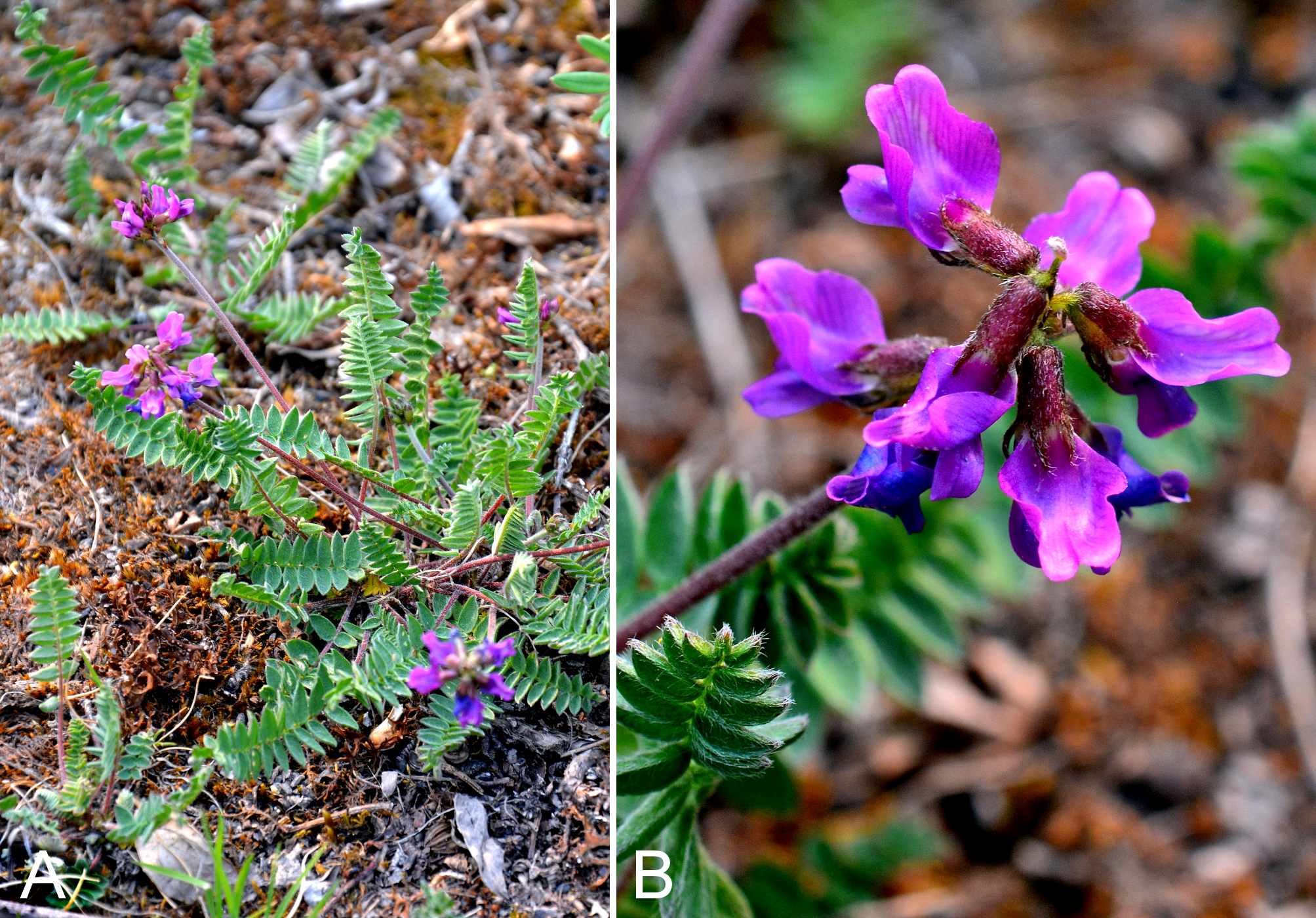

Supplement: Supplemental Information 32 — (A) habit, Saarela et al. 3366. (B) inflorescence, Saarela et al. 3366. Photographs by P. C. Sokoloff. [file peerj-05-2835-s032.png]

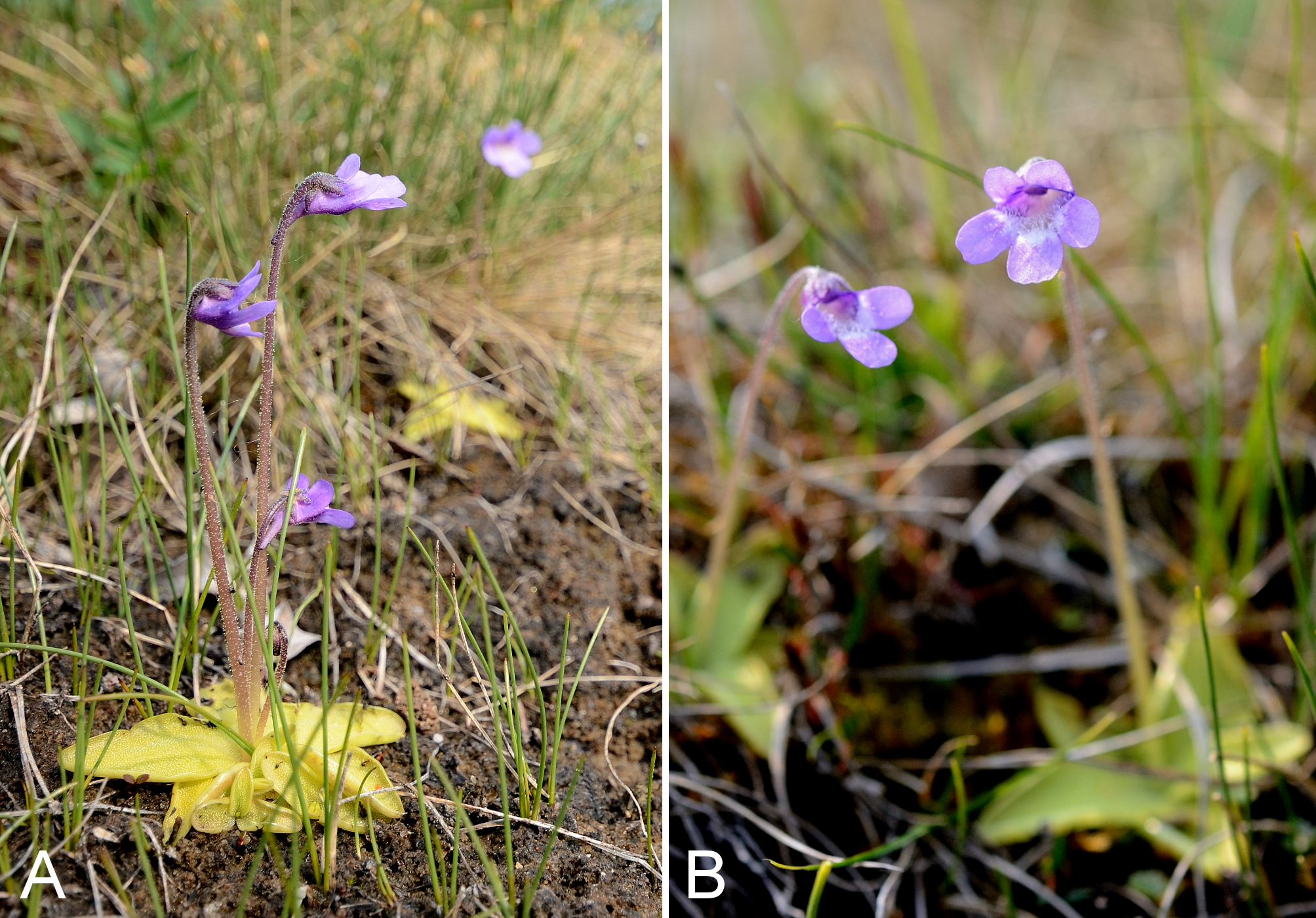

Supplement: Supplemental Information 33 — (A) habit, Saarela et al. 3233. (B) inflorescences, Saarela et al. 3233. Photographs by R. D. Bull. [file peerj-05-2835-s033.png]

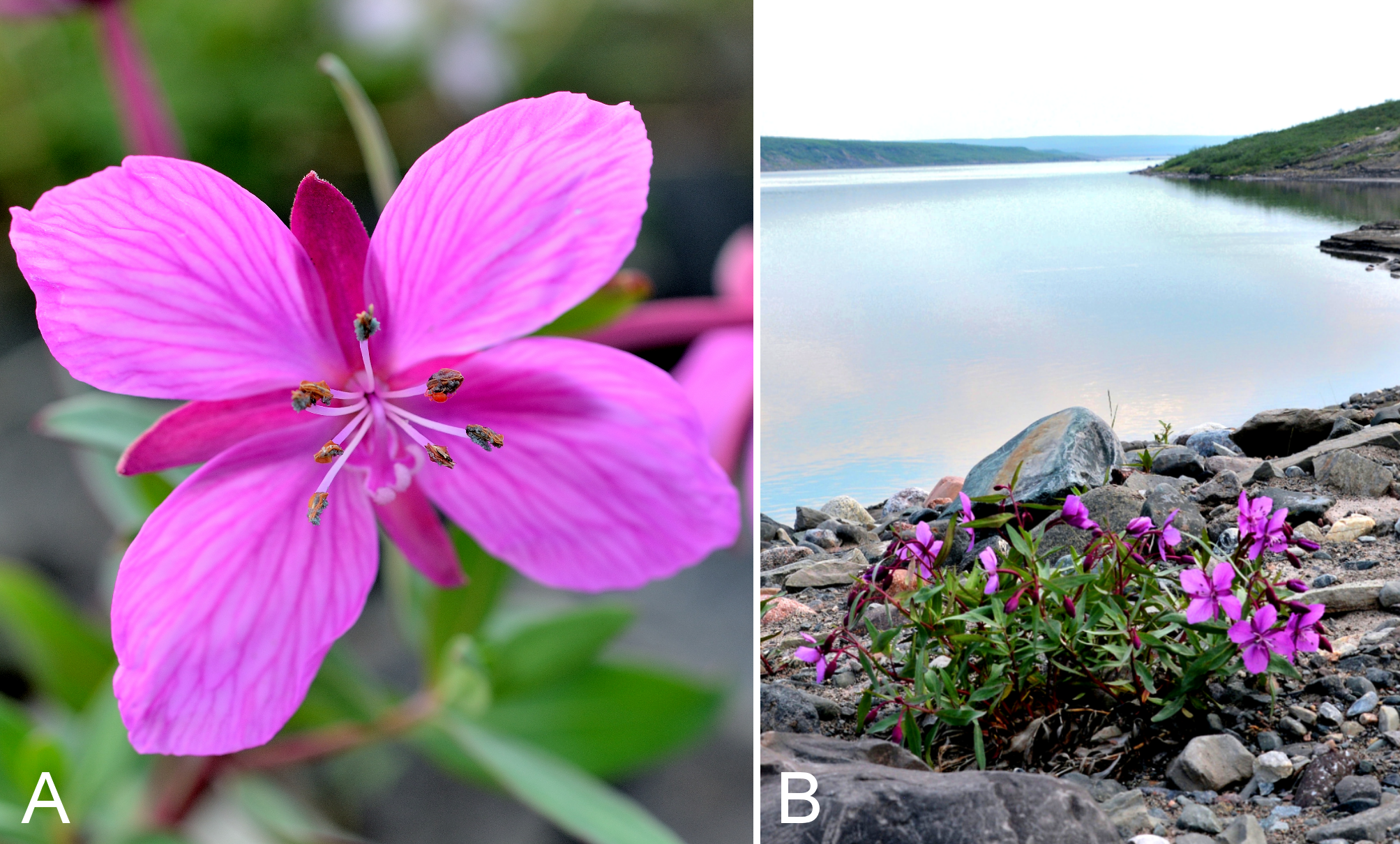

Supplement: Supplemental Information 34 — (A) inflorescence, Saarela et al. 3961. (B) habit, Saarela et al. 3961. Photographs by R. D. Bull (A) and P. C. Sokoloff (B). [file peerj-05-2835-s034.png]

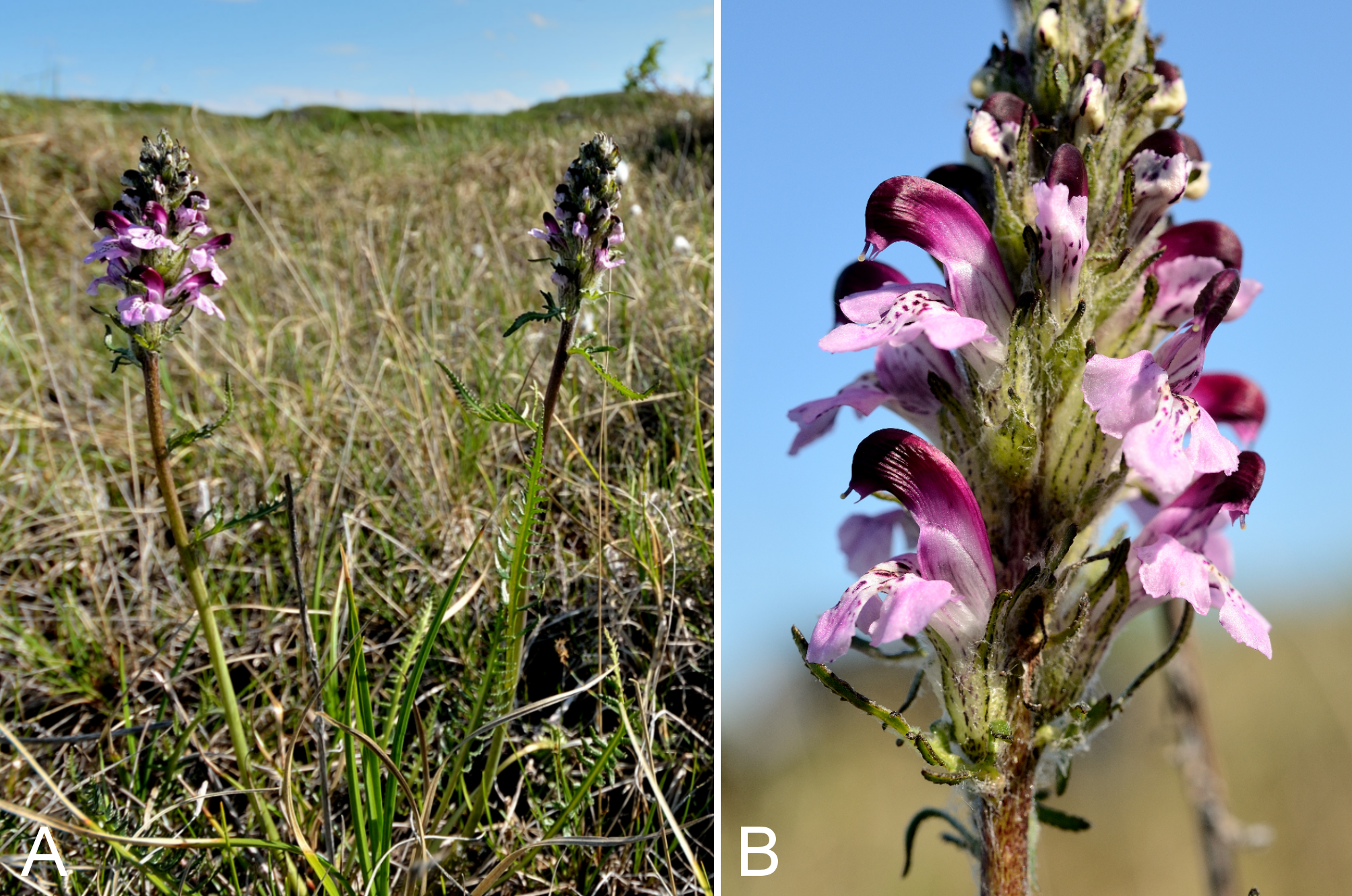

Supplement: Supplemental Information 35 — (A) habit, Saarela et al. 3230a. (B) inflorescence, Saarela et al. 3230a. Photographs by R. D. Bull. [file peerj-05-2835-s035.png]

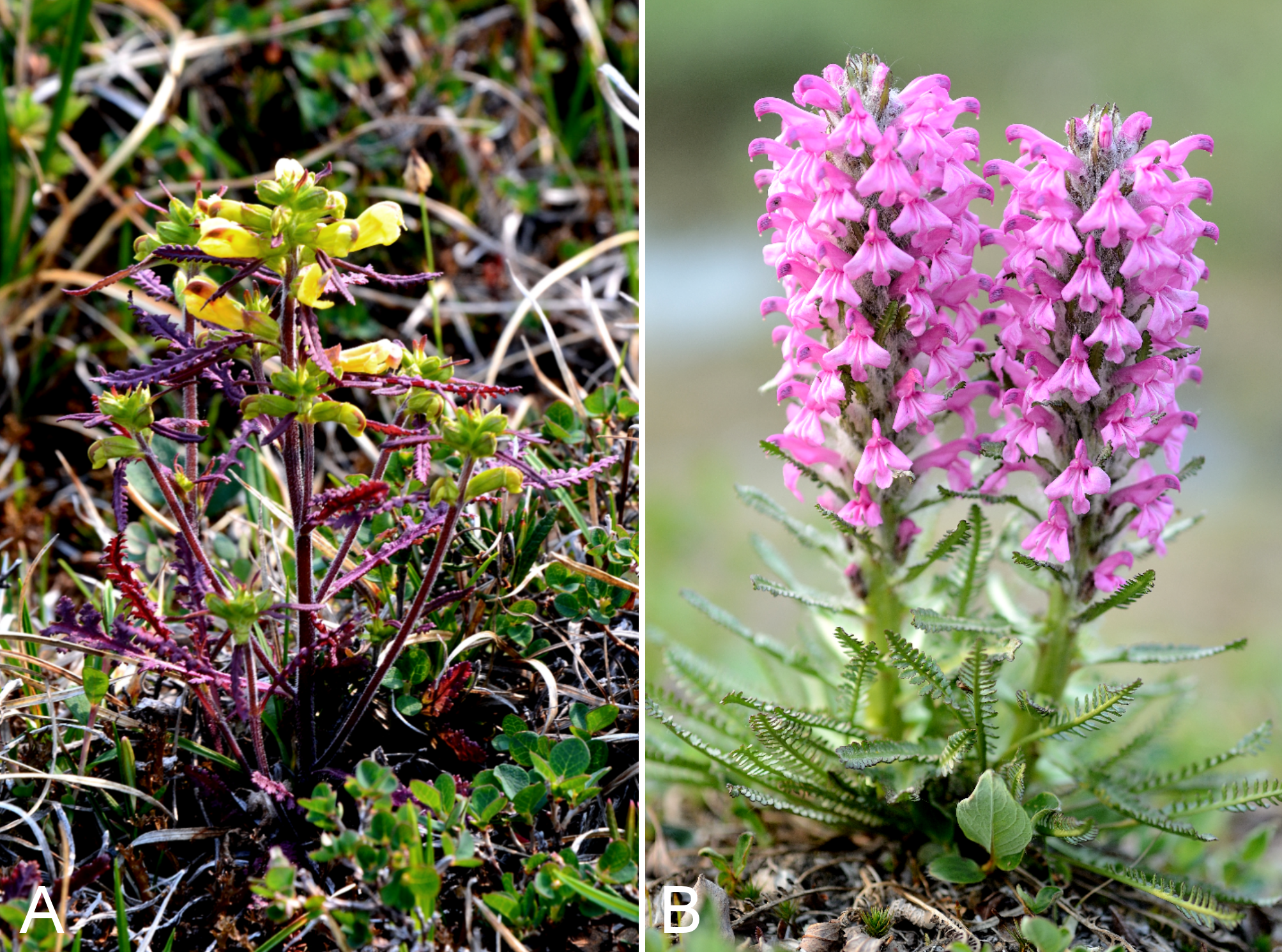

Supplement: Supplemental Information 36 — Pedicularis labradorica: (A) habit, Saarela et al. 3239. Pedicularis lanata: (B) habit, Saarela et al. 3279. Photographs by P. C. Sokoloff (A) and R. D. Bull (B). [file peerj-05-2835-s036.png]

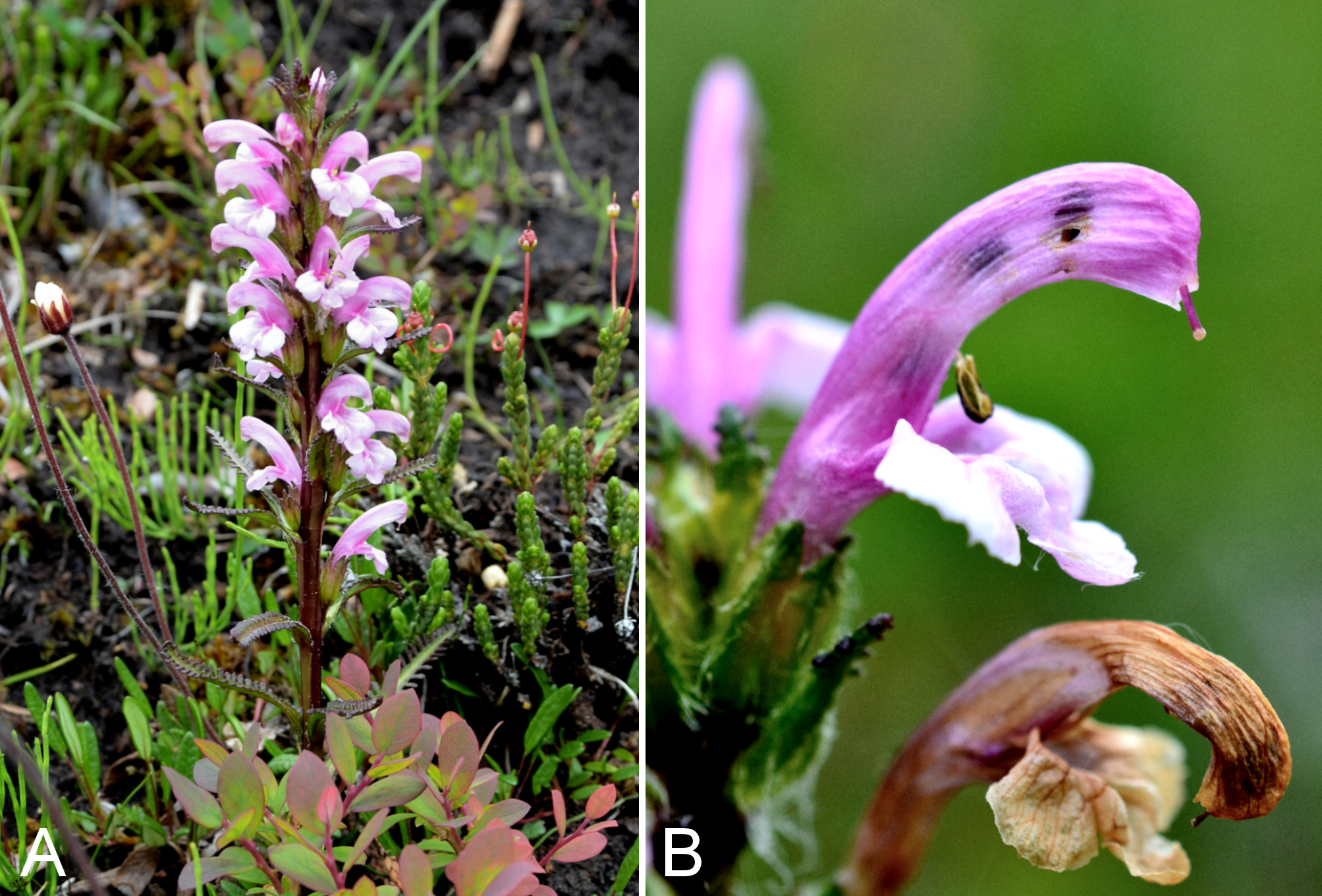

Supplement: Supplemental Information 37 — (A) habit, Saarela et al. 3618. (B) inflorescence, Saarela et al. 3618. Photographs by P. C. Sokoloff. [file peerj-05-2835-s037.png]

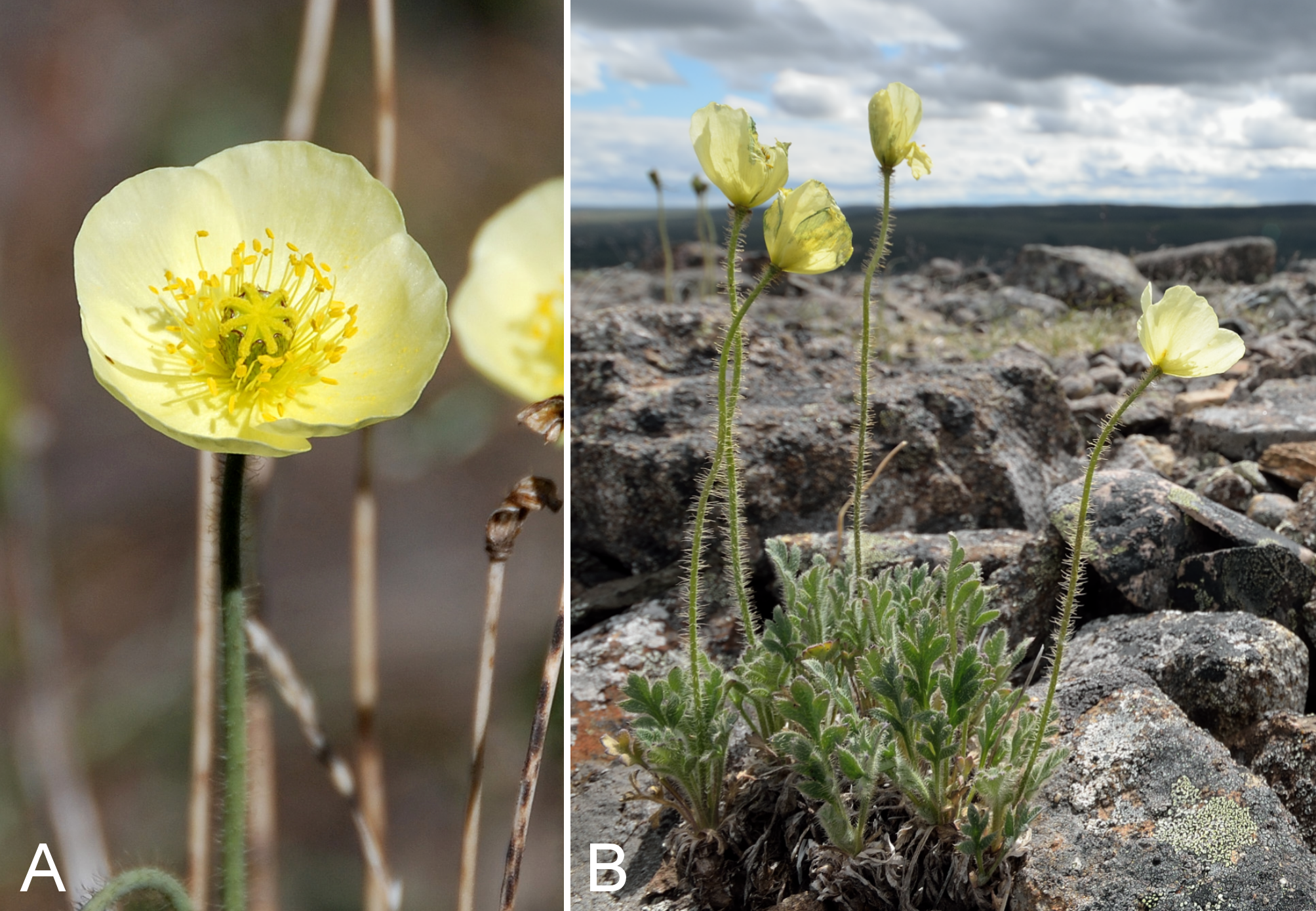

Supplement: Supplemental Information 38 — (A) inflorescence, Saarela et al. 3451. (B) habit, Saarela et al. 3777. Photographs by J. M. Saarela (A) and R. D. Bull (B). [file peerj-05-2835-s038.png]

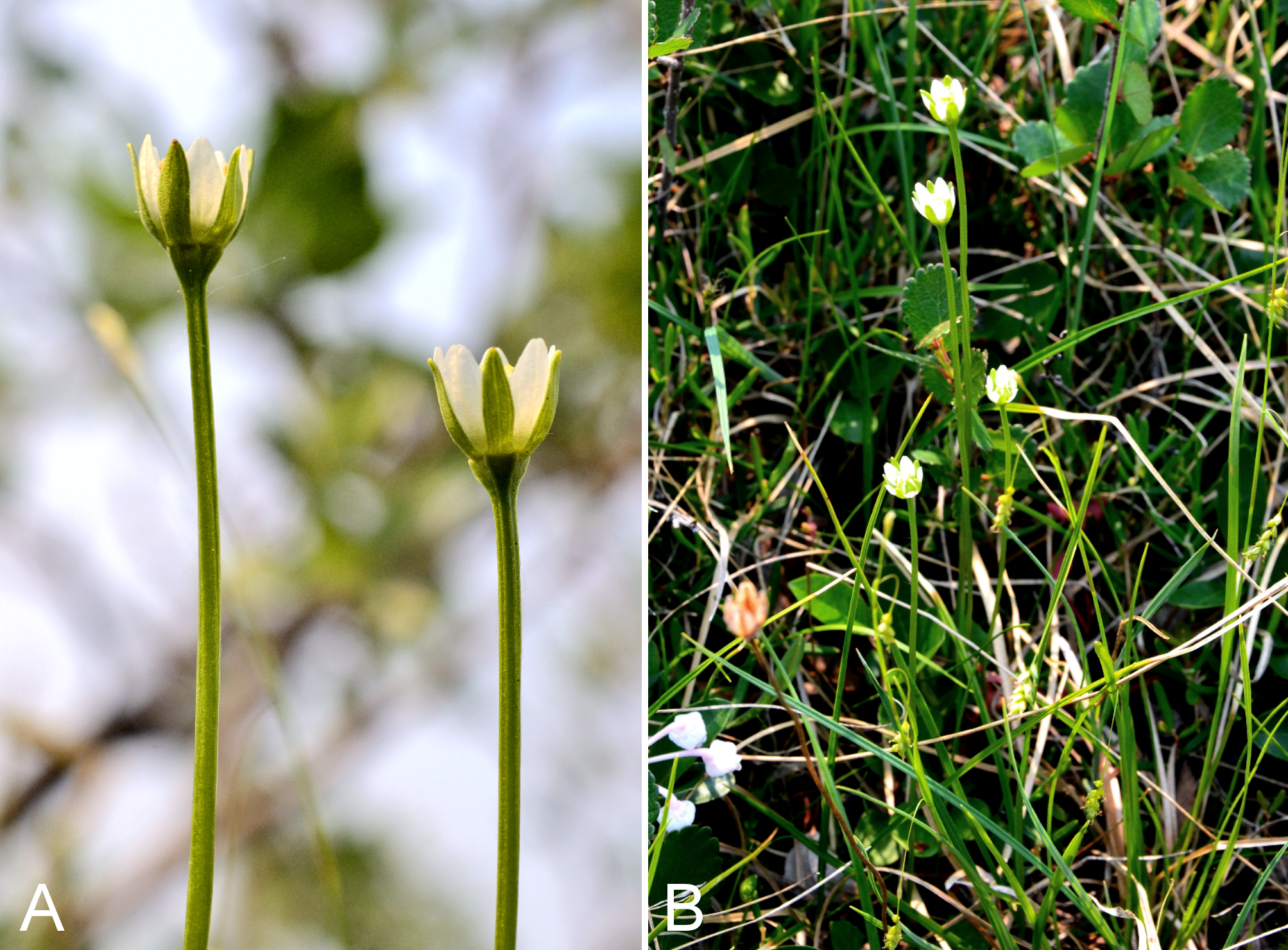

Supplement: Supplemental Information 39 — (A) inflorescences, Saarela et al. 3260. (B) habit, Saarela et al. 3260. Photographs by R. D. Bull (A) and P. C. Sokoloff (B). [file peerj-05-2835-s039.png]

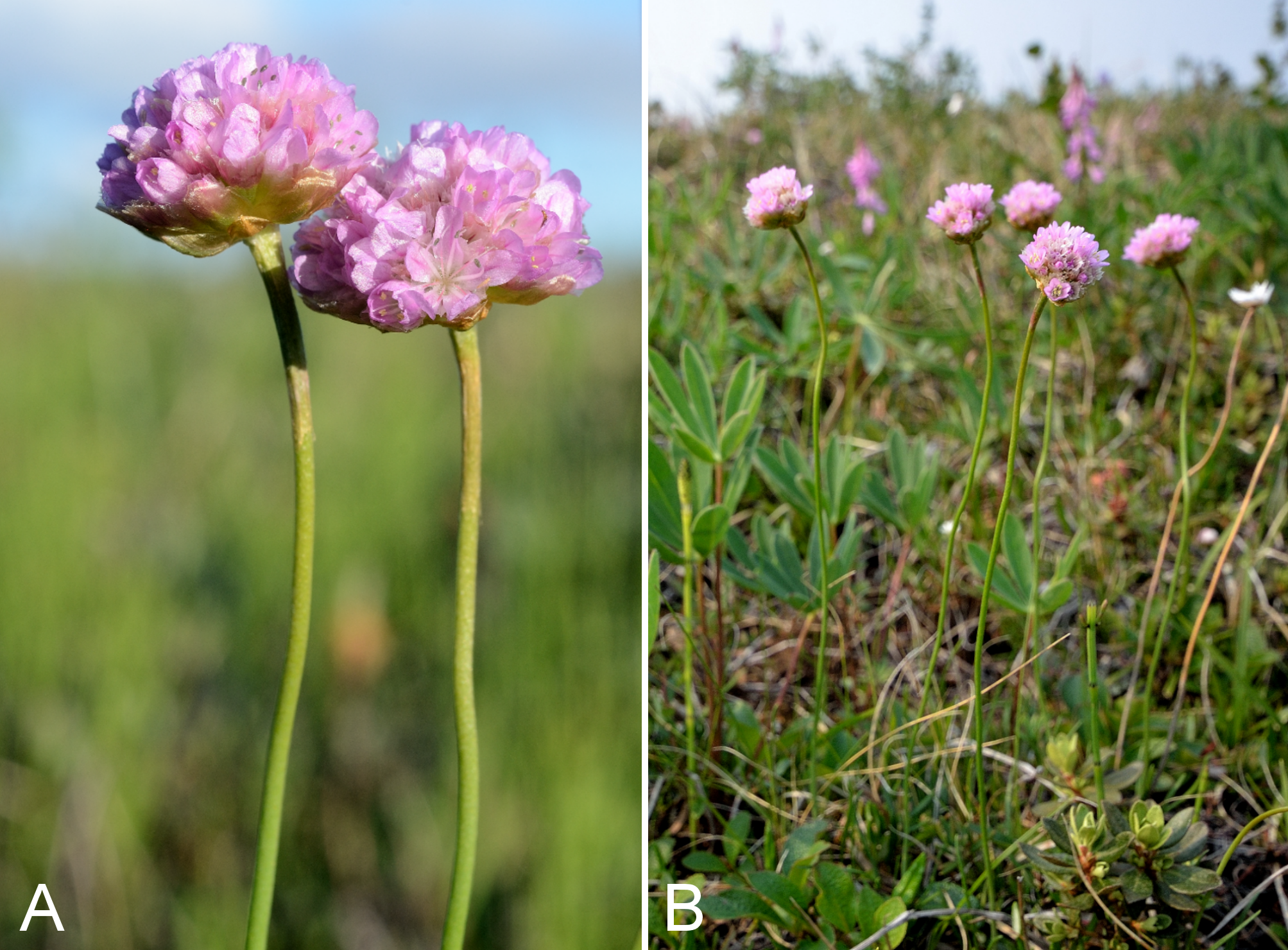

Supplement: Supplemental Information 40 — (A) inflorescence, Kugluk (Bloody Falls) Territorial Park, Nunavut, 16 July 2014. (B) habit, Saarela et al. 3186. Photographs by R. D. Bull. [file peerj-05-2835-s040.png]

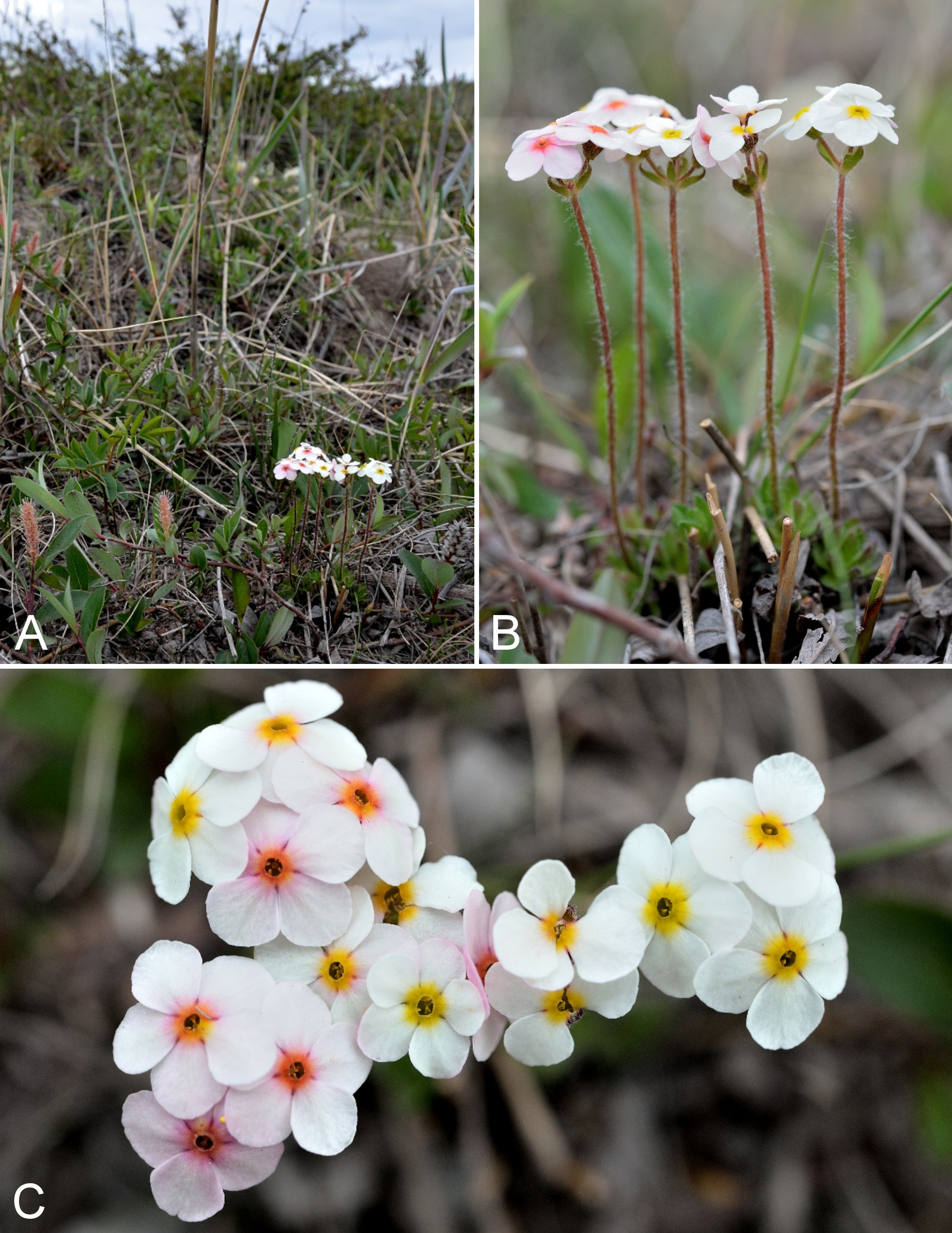

Supplement: Supplemental Information 41 — (A) habitat, Saarela et al. 3679. (B) habit, Saarela et al. 3679. C. inflorescences, Saarela et al. 3679. Photographs by R. D. Bull. [file peerj-05-2835-s041.png]

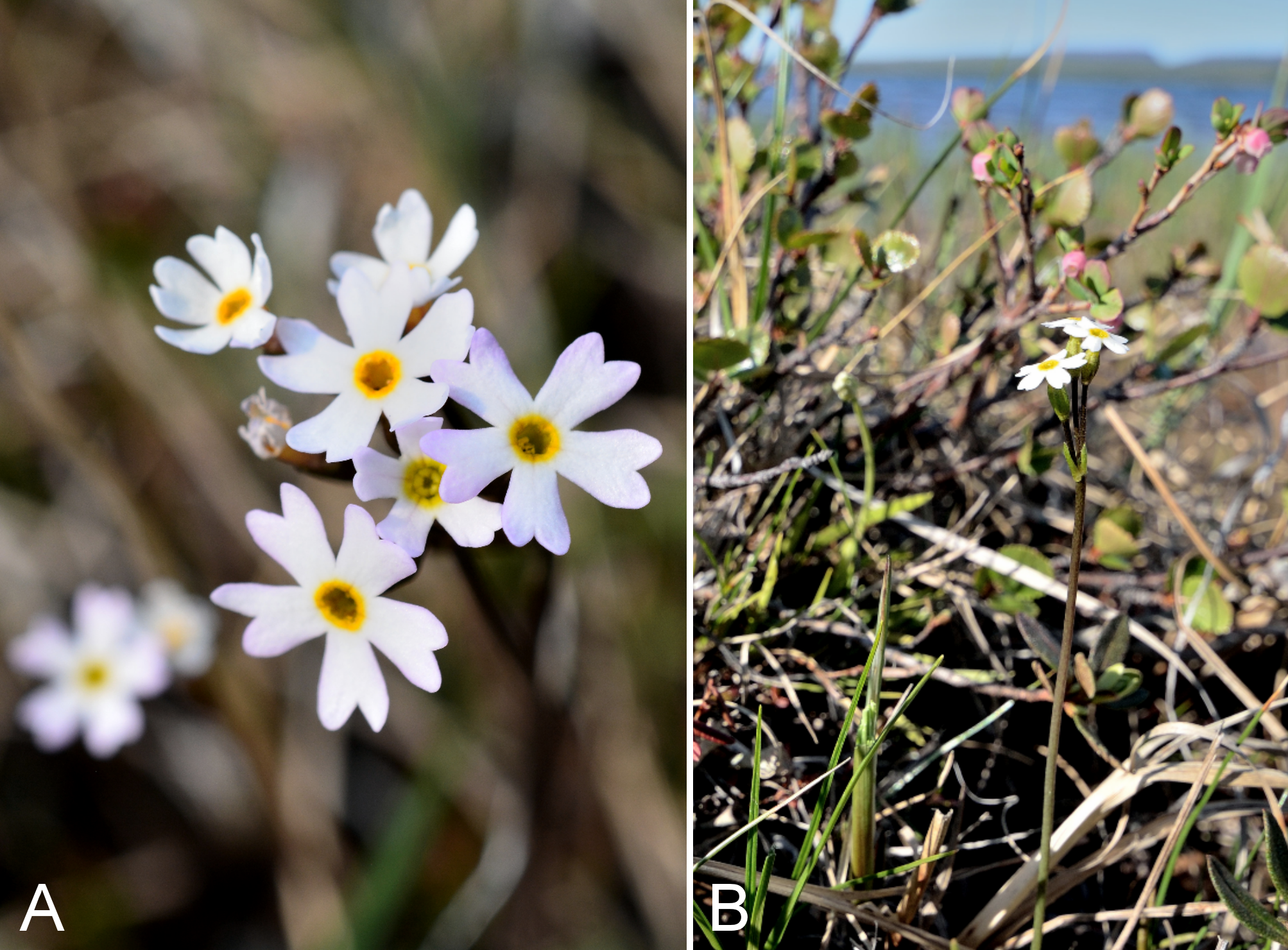

Supplement: Supplemental Information 42 — (A) inflorescences, Saarela et al. 3234. (B) habit, Saarela et al. 3234. Photographs by P. C. Sokoloff (A) and R. D. Bull (B). [file peerj-05-2835-s042.png]

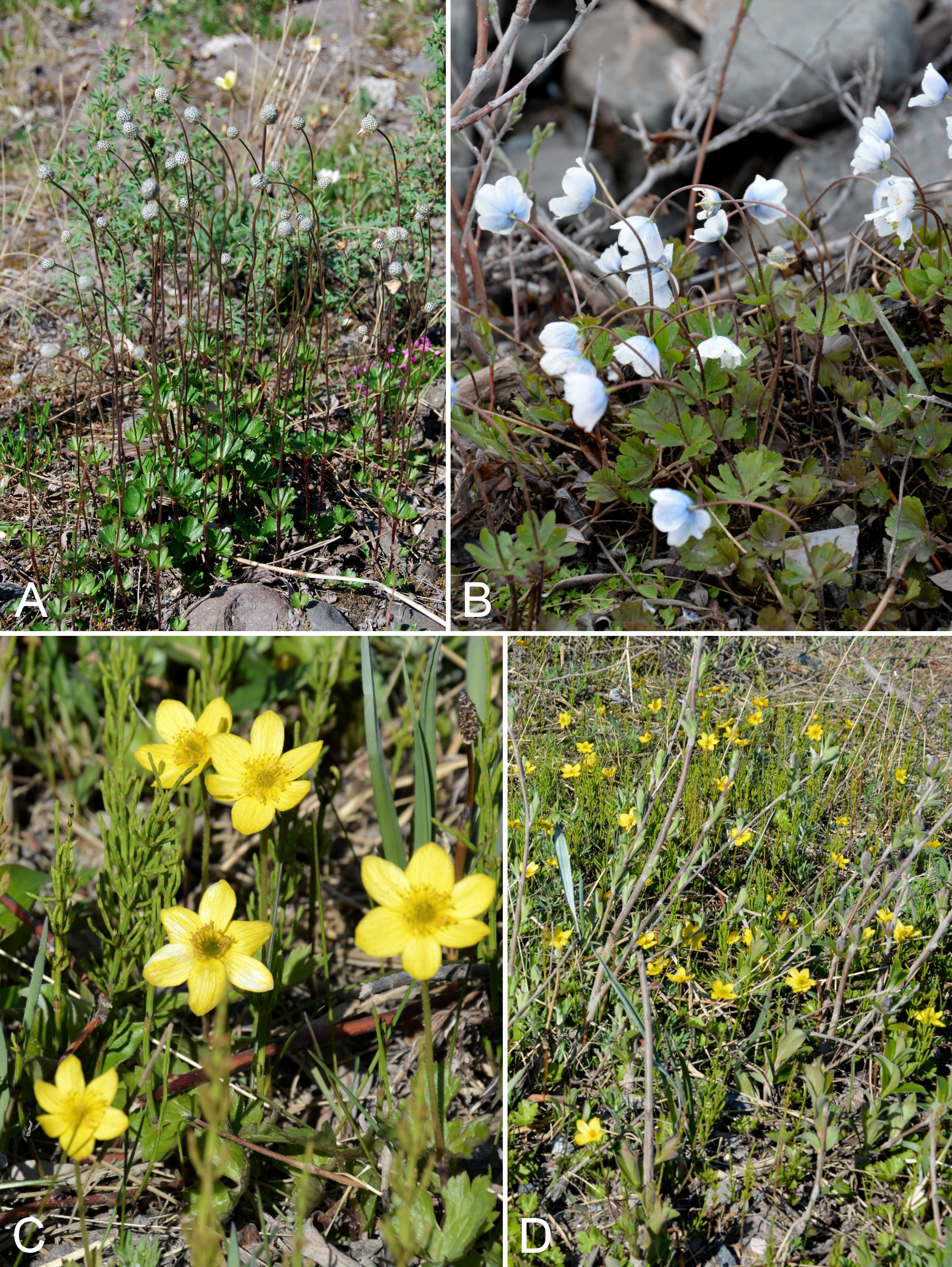

Supplement: Supplemental Information 43 — Anemone parviflora: (A) habit, Saarela et al. 3157. (B) habit, Kugluktuk, Nunavut, 28 June 2014. Anemone richardsonii: (C) habit, Saarela et al. 3103. (D) habitat, Saarela et al. 3103. Photographs by J. M. Saarela (A) and P. C. Sokoloff (B, C, D). [file peerj-05-2835-s043.png]

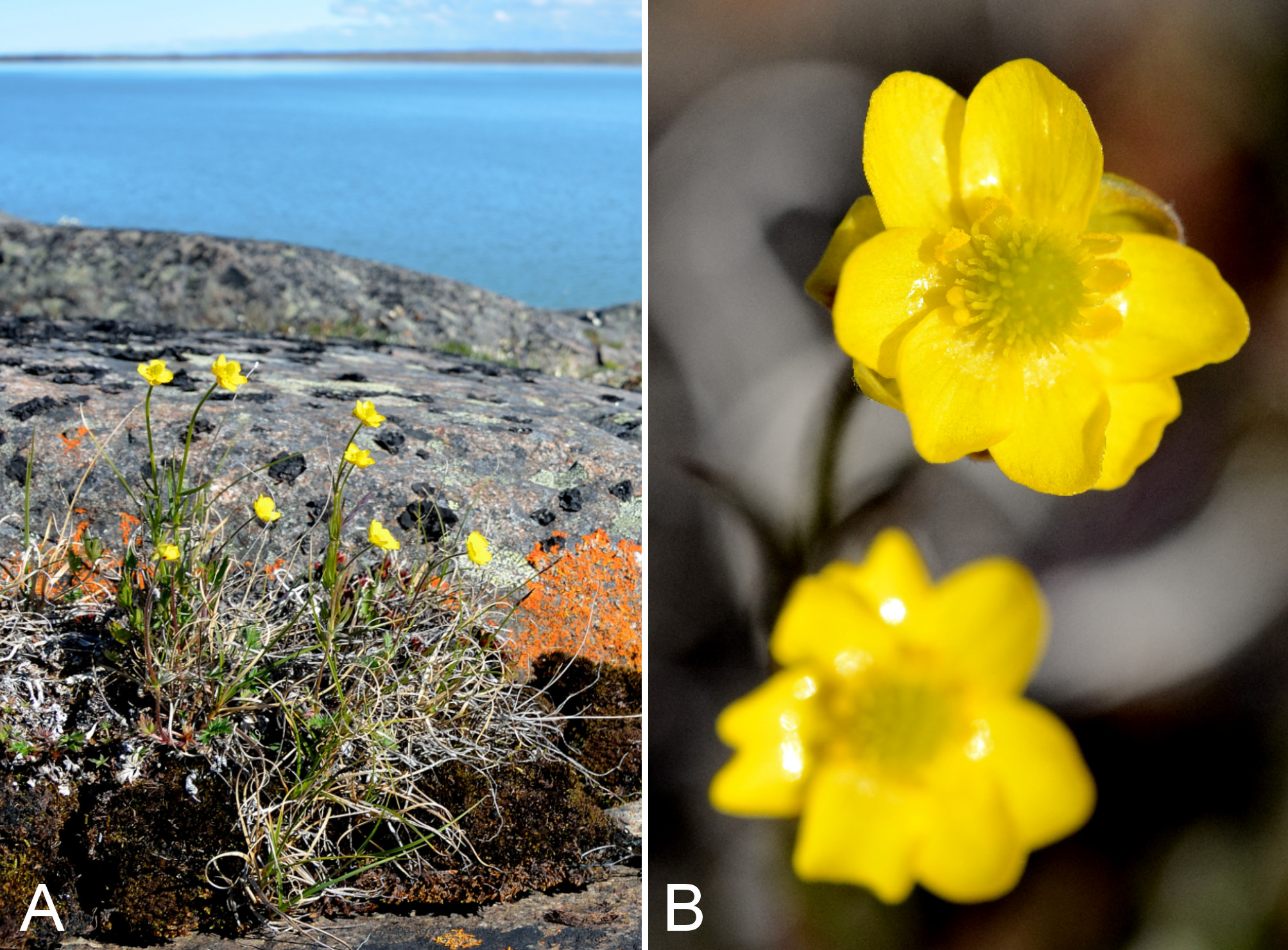

Supplement: Supplemental Information 44 — (A) habit, Saarela et al. 3706. (B) inflorescence, Saarela et al. 3706. Photographs by R. D. Bull. [file peerj-05-2835-s044.png]

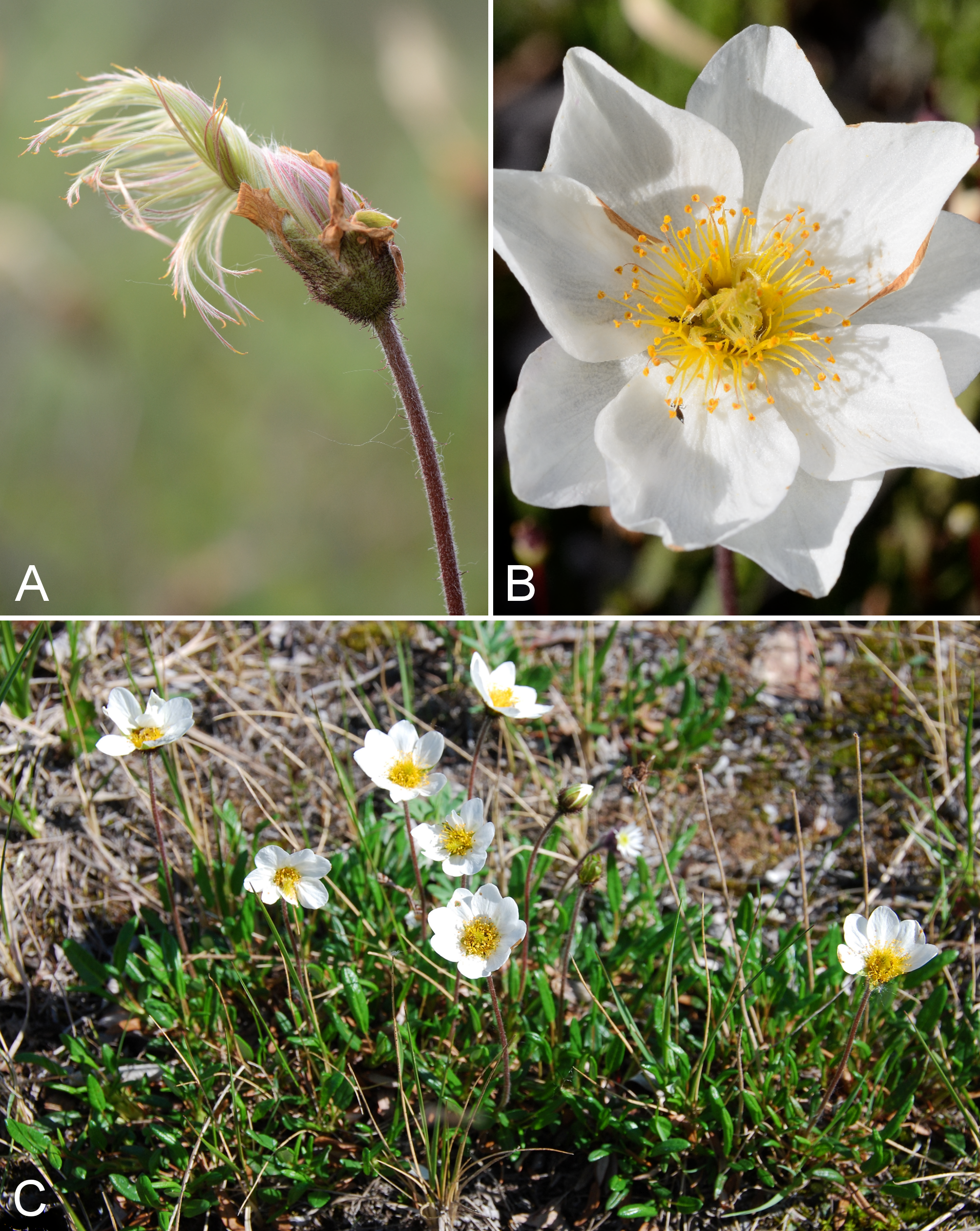

Supplement: Supplemental Information 45 — (A) fruits, vicinity of Fockler Creek, Nunavut, 4 July 2014. (B) inflorescence, Kugluk (Bloody Falls) Territorial Park, Nunavut, 16 July 2014. (C) habit, vicinity of Fockler Creek, Nunavut, 2 July 2014. Photographs by R. D. Bull (A, B) and J. M. Saarela (C). [file peerj-05-2835-s045.png]

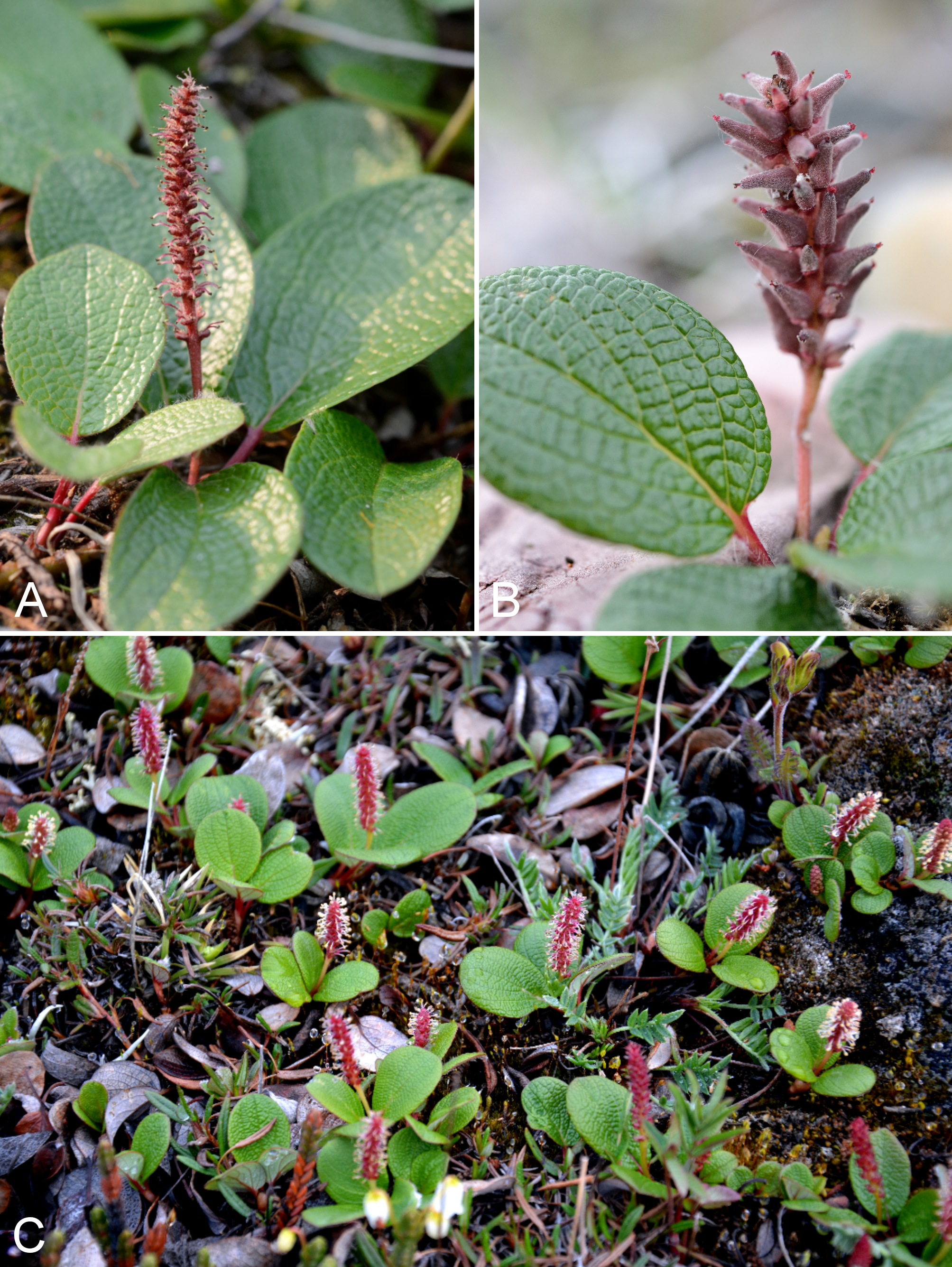

Supplement: Supplemental Information 46 — (A) male catkin, Saarela et al. 3864. (B) female catkin, Kugluk (Bloody Falls) Territorial Park, Nunavut, 13 July 2014. (C) habit, Kugluk (Bloody Falls) Territorial Park, Nunavut, 15 July 2014. Photographs by R. D. Bull (A, B) and P. C. Sokoloff (C). [file peerj-05-2835-s046.png]

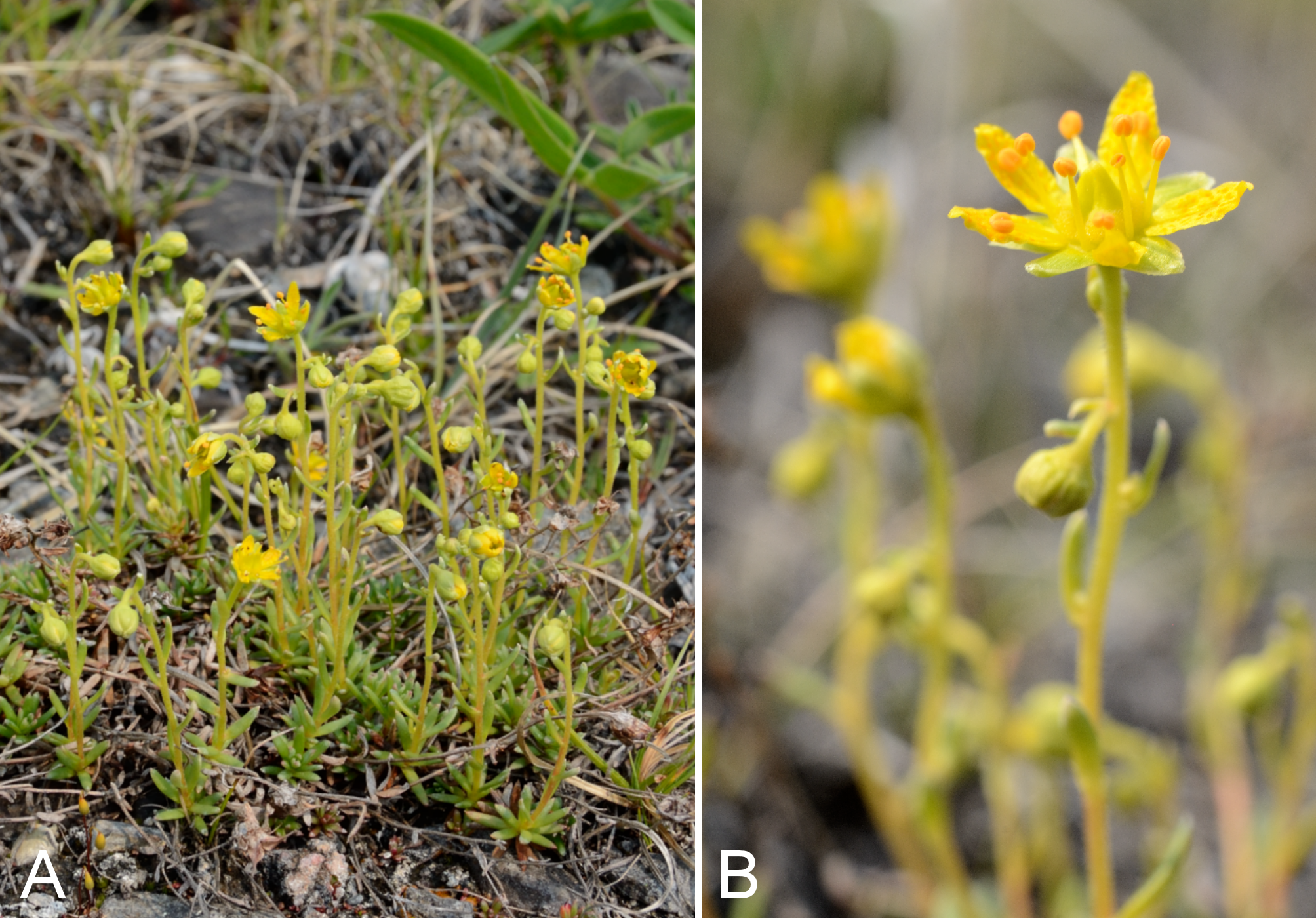

Supplement: Supplemental Information 47 — (A) habit, Saarela et al. 4326. (B) inflorescence, Saarela et al. 4326. Photographs by R. D. Bull. [file peerj-05-2835-s047.png]

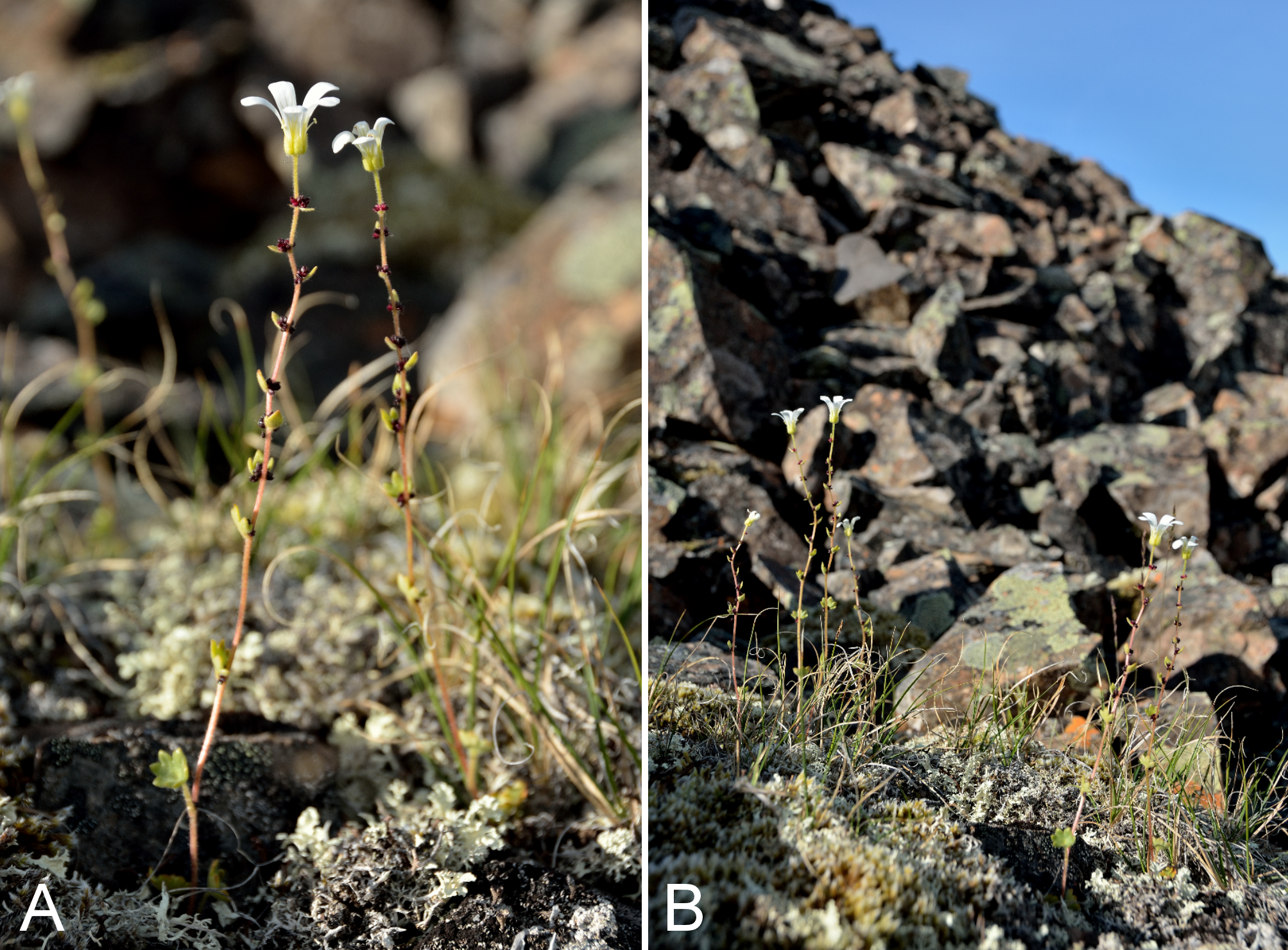

Supplement: Supplemental Information 48 — (A) habit, Saarela et al. 3439. (B) habitat, Saarela et al. 3439. Photographs by R. D. Bull. [file peerj-05-2835-s048.png]

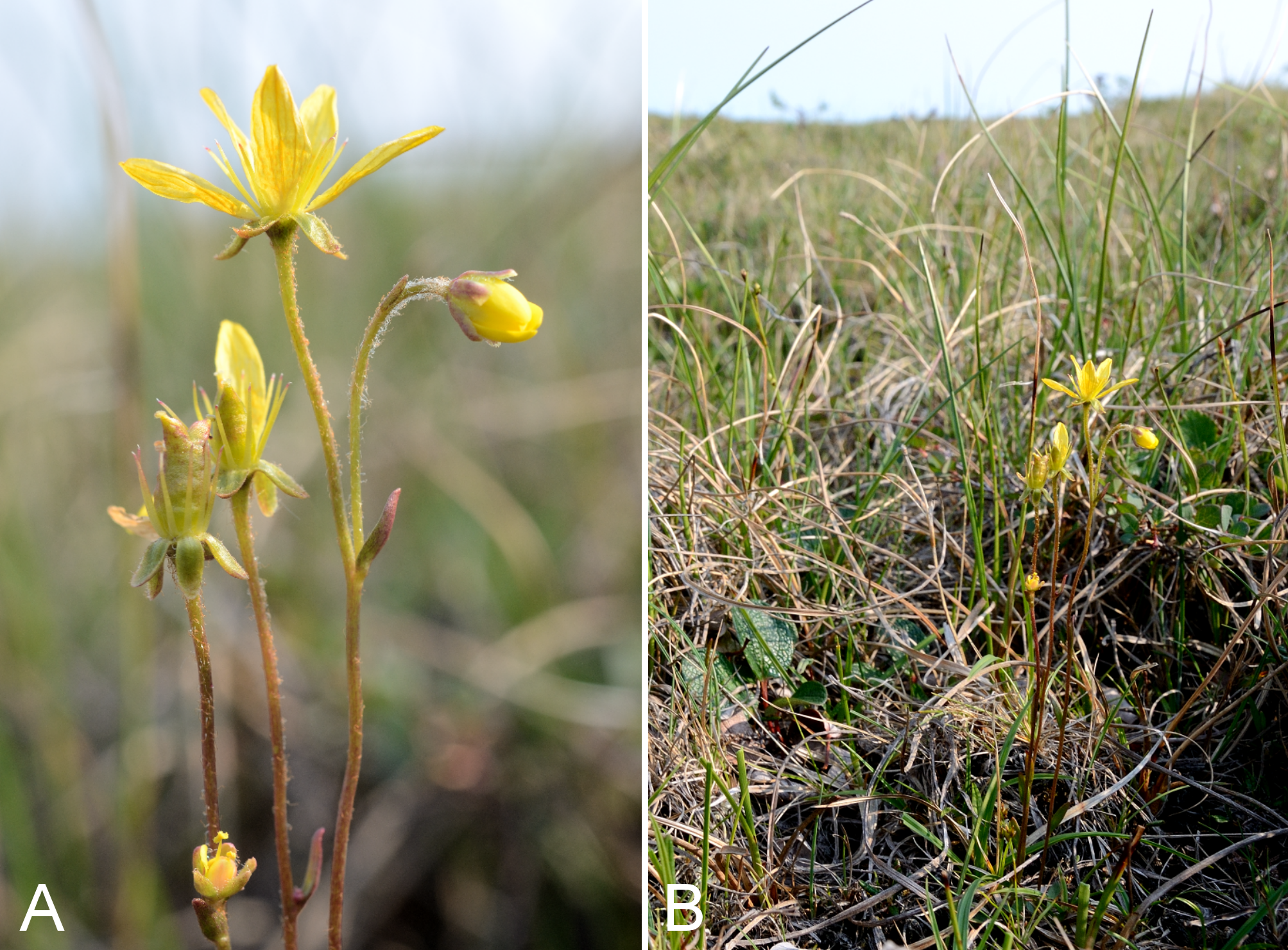

Supplement: Supplemental Information 49 — (A) inflorescence, Saarela et al. 4283. (B) habitat, Saarela et al. 4283. Photographs by R. D. Bull. [file peerj-05-2835-s049.png]

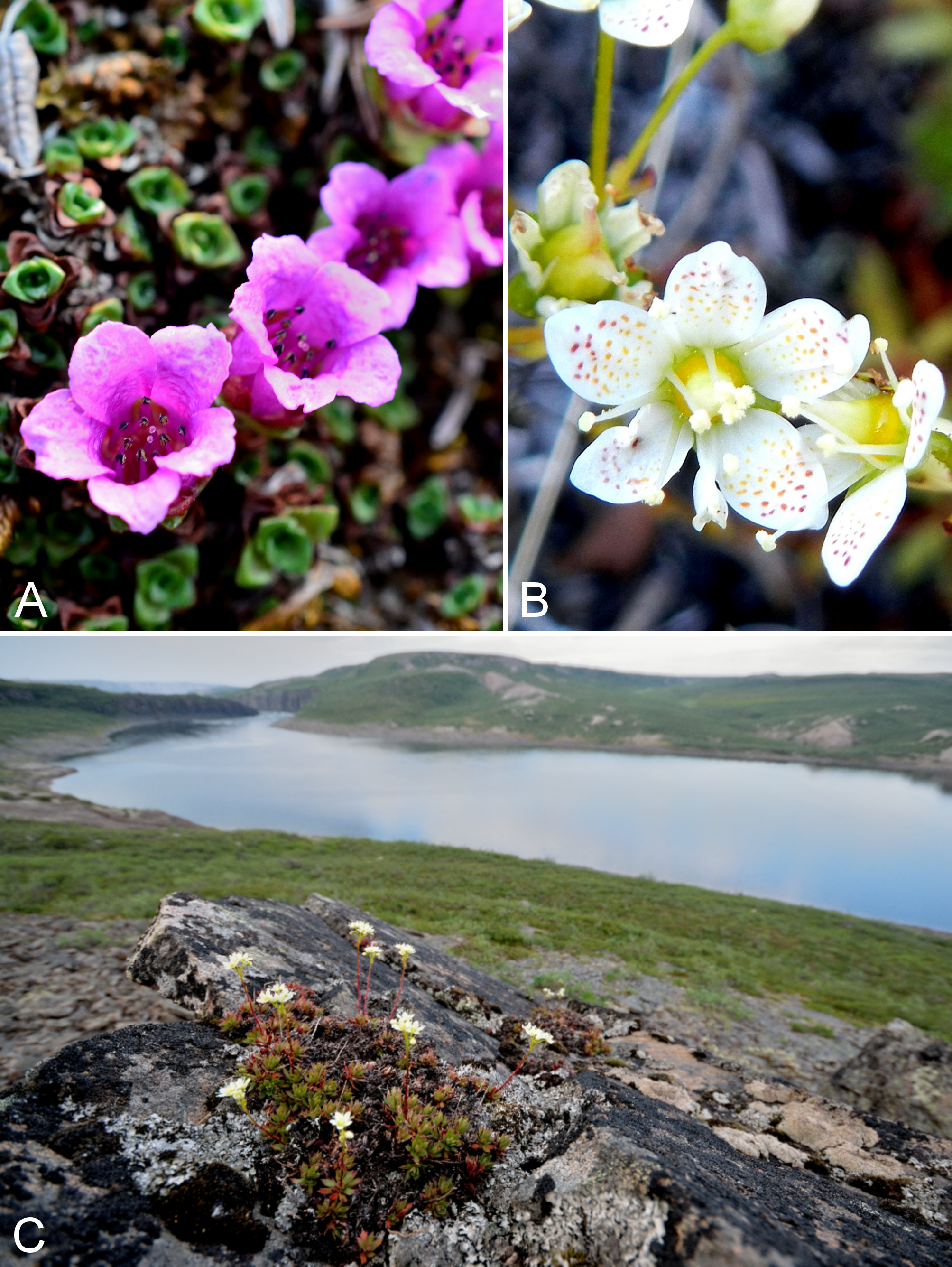

Supplement: Supplemental Information 50 — Saxifraga oppositifolia: (A) inflorescence, Saarela et al. 3280. Saxifraga tricuspidata: (B) inflorescence, vicinity of Fockler Creek, Nunavut, 1 July 2014. (C) habitat, Kugluk (Bloody Falls) Territorial Park, Nunavut, 14 July 2014. Photographs by P. C. Sokoloff (A, B) and R. D. Bull (C). [file peerj-05-2835-s050.png]
